# Overexpression of a truncated CTF7 construct leads to pleiotropic defects in reproduction and vegetative growth in Arabidopsis

**DOI:** 10.1186/s12870-015-0452-2

**Published:** 2015-03-05

**Authors:** Desheng Liu, Christopher A Makaroff

**Affiliations:** Department of Chemistry and Biochemistry, Miami University, Oxford, OH 45056 USA

**Keywords:** Meiosis, Sister chromatid cohesion, Megasporogenesis, Megagametogenesis, Megaspore mother cell, Functional megaspore-like, Epigenetic

## Abstract

**Background:**

Eco1/Ctf7 is essential for the establishment of sister chromatid cohesion during S phase of the cell cycle. Inactivation of Ctf7/Eco1 leads to a lethal phenotype in most organisms. Altering Eco1/Ctf7 levels or point mutations in the gene can lead to alterations in nuclear division as well as a wide range of developmental defects. Inactivation of Arabidopsis *CTF7* (*AtCTF7*) results in severe defects in reproduction and vegetative growth.

**Results:**

To further investigate the function(s) of AtCTF7, a tagged version of AtCTF7 and several AtCTF7 deletion constructs were created and transformed into wild type or *ctf7*^*+/–*^plants. Transgenic plants expressing 35S:NTAP:AtCTF7_∆299–345_ (AtCTF7∆B) displayed a wide range of phenotypic alterations in reproduction and vegetative growth. Male meiocytes exhibited chromosome fragmentation and uneven chromosome segregation. Mutant ovules contained abnormal megasporocyte-like cells during pre-meiosis, megaspores experienced elongated meiosis and megagametogenesis, and defective megaspores/embryo sacs were produced at various stages. The transgenic plants also exhibited a broad range of vegetative defects, including meristem disruption and dwarfism that were inherited in a non-Mendelian fashion. Transcripts for epigenetically regulated transposable elements (TEs) were elevated in transgenic plants. Transgenic plants expressing 35S:AtCTF7∆B displayed similar vegetative defects, suggesting the defects in 35S:NTAP:AtCTF7∆B plants are caused by high-level expression of AtCTF7∆B.

**Conclusions:**

High level expression of AtCTF7∆B disrupts megasporogenesis, megagametogenesis and male meiosis, as well as causing a broad range of vegetative defects, including dwarfism that are inherited in a non-Mendelian fashion.

**Electronic supplementary material:**

The online version of this article (doi:10.1186/s12870-015-0452-2) contains supplementary material, which is available to authorized users.

## Background

The precise establishment and release of sister chromatid cohesion are vital for cell division during mitosis and meiosis [1-3]. The cohesin complex, which forms a ring to entrap sister chromatids, is composed of four subunits: a heterodimer of Structural Maintenance of Chromosome (SMC) proteins, SMC1 and SMC3, an α-kleisin, Sister Chromatid Cohesion1 (SCC1) in somatic cells or REC8 in meiotic cells and SCC3 [2,4,5]. The core cohesin complex associates with a number of factors during the cohesion cycle [1-3,6-8] and its association with the chromosomes is controlled by modifications, such as acetylation, phosphorylation, sumoylation and proteolysis [9-13].

Sister chromatid cohesion is established during S phase by the Establishment of Cohesion protein, ECO1/CTF7 [14-18]. In budding yeast, ECO1/CTF7 acetylates lysine residues K112, K113 of SMC3 and K84, K210 of SCC1 to stabilize the ring and keep it closed [10,19,20]. ECO1/CTF1 is an essential gene in many organisms with the complete inactivation of *CTF7* resulting in lethality [1-3,14-16,21]. Inactivation or mutations in *ECO1/CTF1* leads to a broad range of defects, including chromosome mis-segregation, defects in DNA double strand break repair, defects in homologous recombination and transcriptional alterations [14,15,17,21-25]. Humans contain two ECO1/CTF7 orthologs, *ESCO1* and *ESCO2* [24]. Mutations in *ESCO2* lead to Roberts syndrome (RBS), which is associated with a variety of defects including growth retardation, limb reduction/asymmetric limb growth, cleft lip/palate and missing fingers/toes [26,27].

Arabidopsis contains a single CTF7 ortholog (AtCTF7), which can complement the temperature-sensitive yeast *ctf7-203* mutant [16]. *AtCTF7* encodes a 345 amino acid protein, containing a PIP box at residues 82 to 86, a C_2_H_2_ zinc finger motif at residues 92–130 and an acetyltransferase domain from residues 184 to 335. The acetyltransferase domain can be further separated into three motifs: D (amino acids 184–204), A (amino acids 266–301) and B (amino acids 311–335). Motif D provides the framework of the acetyltransferase domain. Motif A participates in acetyl-CoA binding and is critical for catalytic activity, while motif B is the most C-terminal region and participates in substrate recognition and catalytic activity regulation [28,29]. Variations in *AtCTF7* expression have been shown to result in a wide range of defects. Plants heterozygous for a T-DNA insertion in *AtCTF7* grow normally but their siliques contain approximately 25% arrested seeds, consistent with the belief that CTF7 is an essential protein [16]. However, *Atctf7* homozygous plants can be detected at very low frequencies [17]. *Atctf7* plants exhibit a wide range of developmental defects, including extreme dwarfism and sterility with *Atctf7* meiocytes exhibiting abnormal chromosome segregation and defective sister chromatid cohesion. Knockdown of *AtCTF7* mRNA levels using RNAi leads to growth retardation and defective sister chromatid cohesion [18]. Finally, overexpression of full length AtCTF7 with the CaMV 35S promoter leads to ovule abortion, but plant growth is not affected [16].

To further investigate the roles of CTF7 in Arabidopsis and to identify AtCTF7 interacting proteins, transgenic plants expressing AtCTF7 constructs that encode either TAP-tagged [30] or non-tagged versions of full length or truncated AtCTF7 were generated and analyzed. Transgenic plants that express a truncated version of AtCTF7, missing motif B, from the 35S promoter exhibited impaired reproduction and vegetative growth. Ovules in 35S:NTAP:AtCTF7∆B plants displayed alterations in cell identity and the timing of megasporogenesis and megagametogenesis, while male meiocytes exhibited alterations during meiosis II. The transgenic plants also displayed alterations in vegetative growth that were not inherited in a Mendelian fashion.

## Results

### Arabidopsis plants expressing high levels of NTAP:AtCTF7∆B display reduced fertility

**Figure 1:**
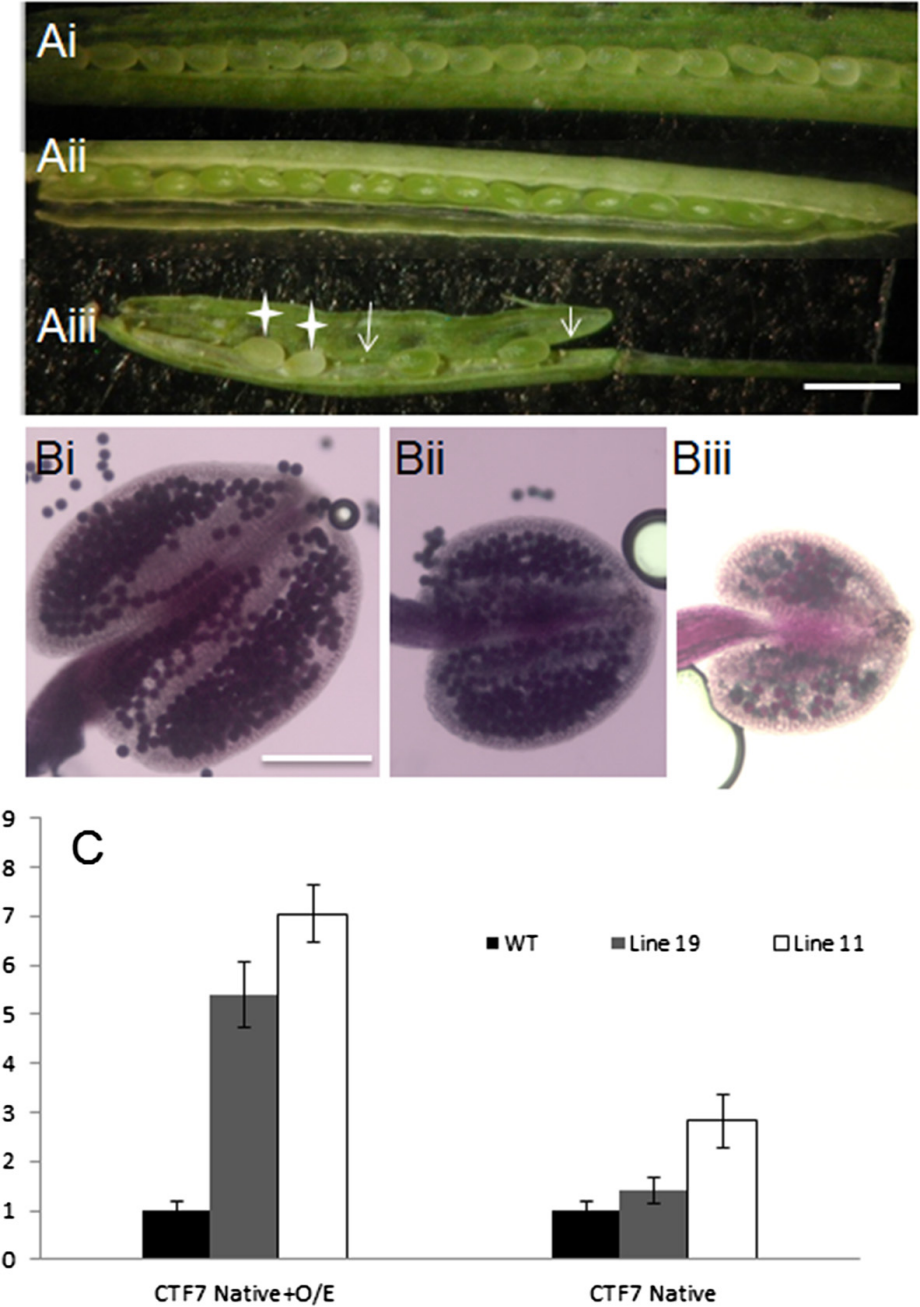
**35S:NTAP:AtCTF7∆B plants exhibit reduced fertility. (A)** Open siliques from wild type **(Ai)** and 35S:NTAP:AtCTF7∆B **(Aii,Aiii)** plants. **i**, Wild type silique with full seed set. **ii**, Silique from a 35S:NTAP:AtCTF7∆B plant (Line 19) exhibiting a weak phenotype. **iii**, Silique from a 35S:NTAP:AtCTF7∆B plant (Line 11) exhibiting a strong phenotype. Arrows indicate shriveled, unfertilized ovules. White stars show white, plump seeds, which aborted after fertilization. Scale bar = 0.5 cm. **(B)** Alexander staining of mature anthers from wild type **(i)** and 35S:NTAP:AtCTF7∆B **(ii, iii)** plants. **i**, Wild type anther. **ii**, Anther from Line 19. The anther is smaller and contains less pollen; all the pollen is viable. **iii**, Anther from Line 11. The anther is smaller and contains low numbers of viable pollen. Scale bar = 50 μm. **(C)**. Expression analysis of *AtCTF7* in wild type and 35S:NTAP:AtCTF7∆B plants. Transcript levels of total *AtCTF7* (35S:NTAP:AtCTF7∆B and native AtCTF7) and native *AtCTF7* are increased in 35S:NTAP:AtCTF7∆B plants with Line 11 plants exhibiting the highest levels. Buds of wild type, non-dwarf, 4th generation Line 11 plants and 4th generation Line19 plants were used for this experiment. Data are shown as means ± SD (n = 3).

An *AtCTF7* construct missing the C-terminal 46 amino acids (∆299-345) was generated, fused with NTAP [30] and expressed from the CaMV 35S promoter in wild-type Columbia plants (35S:NTAP:AtCTF7∆B; Additional file 1: Figure S1). Twenty out of the 36 independent lines examined exhibited reduced fertility, with fertility levels varying significantly between the lines. Plants exhibiting a weak phenotype, which accounted for two of the 20 reduced fertility lines, produced shorter siliques with reduced numbers of seeds, but the seeds appeared normal (Figure 1Aii). For example Line 19 produced 39.2 ± 3.9 seeds per silique (n = 35) compared to wild type plants that produce 54.2 ± 4.1 seeds per silique (n = 35). Anthers from Line 19 plants were smaller and contained reduced numbers of pollen (574 vs 1175 in wild type), but the pollen appeared viable (Figure 1Bii). The other 18 reduced fertility lines exhibited more severe defects. These plants produced siliques containing large numbers of unfertilized ovules and aborted seeds (Figure 1Aiii). Unfertilized ovules appeared as white dots, resembling the situation in *atctf7-1* plants [17]. Aborted seeds appeared white and plump, similar to seeds containing arrested embryos in *Atctf7-1*^*+/−*^ plants [16]. Seed set varied considerably between the lines, ranging from 13.3 ± 5.6 seeds per silique to 34.7 ± 8.4 seeds per silique. Anthers from these plants typically contained reduced numbers of pollen, much of which was not viable. For example, anthers from Line 11 produced on average approximately 210 pollen, of which only 20% was viable (Figure 1Biii). Given that most lines exhibited severe fertility defects, one representative line (#11) was chosen and characterized in detail.

The reduced fertility phenotype of 35S:NTAP:AtCTF7∆B plants suggested that co-suppression may be lowering *AtCTF7* transcript levels in the lines. Therefore, quantitative reverse transcription polymerase chain reaction (qRT-PCR) was conducted to examine transcript levels of native *AtCTF7* as well as the *CTF7* transgene. Surprisingly, total *AtCTF7* (35S:NTAP:AtCTF7∆B and native *AtCTF7*) transcript levels were approximately seven-fold higher in 35S:NTAP:AtCTF7∆B plants than *CTF7* transcript levels in wild type plants, while native *AtCTF7* transcript levels were almost three-fold higher in the transgenic plants (Figure 1C). Therefore, the transgenic lines contained high levels of *AtCTF7∆B* transcripts along with elevated levels of native *AtCTF7* mRNA, indicating that the observed phenotypes are not the result of reduced *AtCTF7* expression. Instead these results suggest that the 35S:NTAP:AtCTF7∆B construct may be exerting a dominant negative effect.

Reciprocal crossing experiments were carried out to determine the effect of the 35S:NTAP:AtCTF7∆B construct on male and female gametes. The 35S:NTAP:AtCTF7∆B construct was transmitted at reduced levels through both male and female gametes (Additional file 1: Table S1), consistent with our preliminary results that both male and female fertility were affected in 35S:NTAP:AtCTF7∆B plants. Nonviable seeds were obtained when 35S:NTAP:AtCTF7∆B plants were used as either the male or female parent; however the relative proportion of nonviable seed was three times greater (9.3% verses 31.3%) when 35S:NTAP:AtCTF7∆B plants served as the female. The number of nonviable seeds was greatest (65%) in self-pollenated plants. Therefore, while both male and female gametes are affected, the 35S:NTAP:AtCTF7∆B construct has a greater effect on female reproduction. Inheritance of 35S:NTAP:AtCTF7∆B-associated defects from both parents has a compounding effect on seed production.

### Male meiotic chromosome segregation is altered in 35S:NTAP:AtCTF7∆B plants

Previous studies showed that inactivation of *AtCTF7* via T-DNA insertion or reduction in *AtCTF7* mRNA levels via RNAi disrupts sister chromatid cohesion, resulting in uneven chromosome segregation during meiosis and ultimately reduced pollen viability [17,18]. The reduced fertility observed in 35S:NTAP:AtCTF7∆B plants suggested that meiosis might also be affected in these lines. Therefore, chromosome spreading experiments were carried out to examine the effect of 35S:NTAP:AtCTF7∆B expression on male meiosis.

**Figure 2:**
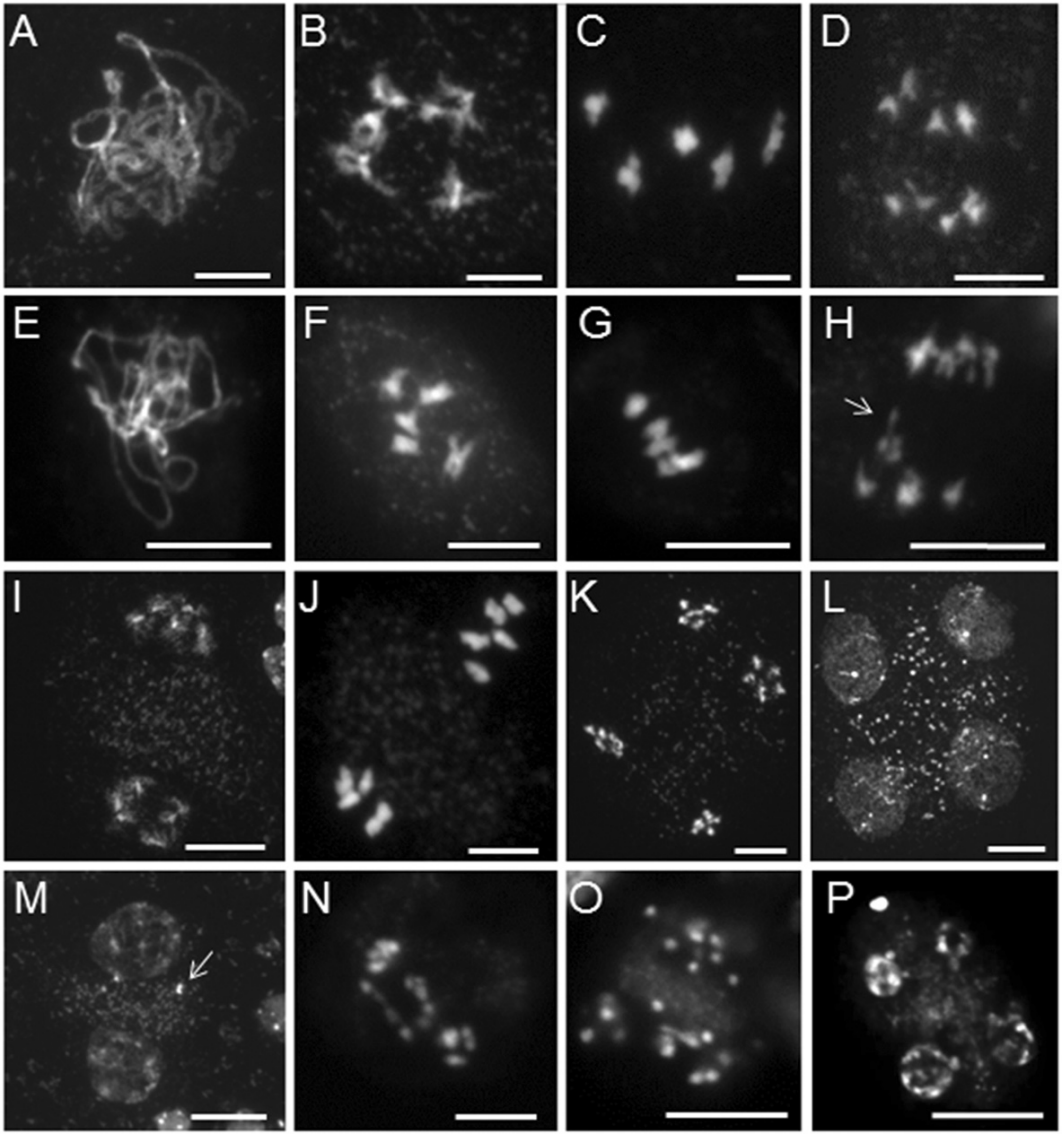
**35S:NTAP:AtCTF7∆B male meiocytes exhibit defective meiotic chromosome segregation. (A-D)** and **(I-L)** Wild type meiocytes. **(E-H)** and **(M-P)** 35S:NTAP:AtCTF7∆B meiocytes. **A**, **E** pachytene; **B**, **F** diakinesis; **C**, **G** metaphase I; **D**, **H** telophase I; **I**, **M** prophase II; **J**, **N** metaphase II; **K**, **O** anaphase II; **L**, **P** telophase II. Lagging chromosomes and/or chromosome fragments are denoted with arrows. Meiotic chromosomes are stained by 4’, 6-diamidino-2-phenylindole (DAPI). Scale bar = 10 μm.

Male meiocytes in 35S:NTAP:AtCTF7∆B plants resembled wild type during early stages of meiosis, with normal chromosome morphology during pachytene (Figure 2A,E), diakinesis (Figure 2B,F) and metaphase I (Figure 2C,G). The first noticeable defect was observed at telophase I when lagging chromosomes were observed (Figure 2D,H), followed by mis-segregated chromosomes at prophase II (Figure 2M). More than twenty individual chromosomes were typically observed in meiocytes beginning at metaphase II (Figure 2N), indicating that sister chromatid cohesion was prematurely lost. Chromosomes did not segregate evenly at anaphase II (Figure 2O) resulting in the production of polyads with varying DNA contents at tetrad stage (Figure 2P). Similar to *atctf7* plants, a small number of relatively normal meiocytes were also observed throughout meiosis with fewer normal-appearing meiocytes in later stages of meiosis. For example, the percentages of defective meiocytes observed at various stages of meiosis in Line 11 were: metaphase I: 0% (0/41), telophase I: 6.8% (7/87), prophase II: 23.5% (24/102), metaphase II: 40.7% (24/59), anaphase II: 56.8% (25/44) and telophase II: 64.0% (114/178).

Immunolocalization experiments were then carried out to examine the distribution of the meiotic cohesin protein SYN1 [31] in 35S:NTAP:AtCTF7∆B male meiocytes. Consistent with meiotic chromosome spreading experiments the overall distribution of SYN1 was not dramatically affected in meiocytes of 35S:NTAP:AtCTF7∆B plants (Additional file 1: Figure S2). SYN1 exhibited diffuse nuclear labeling during interphase with strong signal observed on the developing chromosomal axes from early leptotene into zygotene (Additional file 1: Figures S2A,D). During late zygotene and pachytene the protein lined the chromosomes (Additional file 1: Figures S2B,E,C and F). SYN1 was released normally from the condensing chromosomes during diplotene and diakinesis and similar to the situation in wild type, SYN1 was barely detectable on chromosomes by prometaphase I. Therefore, cohesin appears to load and be removed normally from meiotic chromosomes in 35S:NTAP:AtCTF7∆B plants.

### Female gametophyte development is altered in 35S:NTAP:AtCTF7∆B plants

Previous studies showed that ovule development is very sensitive to *AtCTF7* levels [16-18]. Approximately 50% of the ovules from *Atctf7-1*^+/–^plants contain non-degenerated antipodal nuclei [16], while ovules from *atctf7* plants degenerate early [17]. RNAi directed reduction of *AtCTF7* mRNA levels, as well as 35S-mediated increases in *AtCTF7* transcript levels result in ovule arrest at Female Gametophyte (FG) 1 stage [16,18].

**Figure 3:**
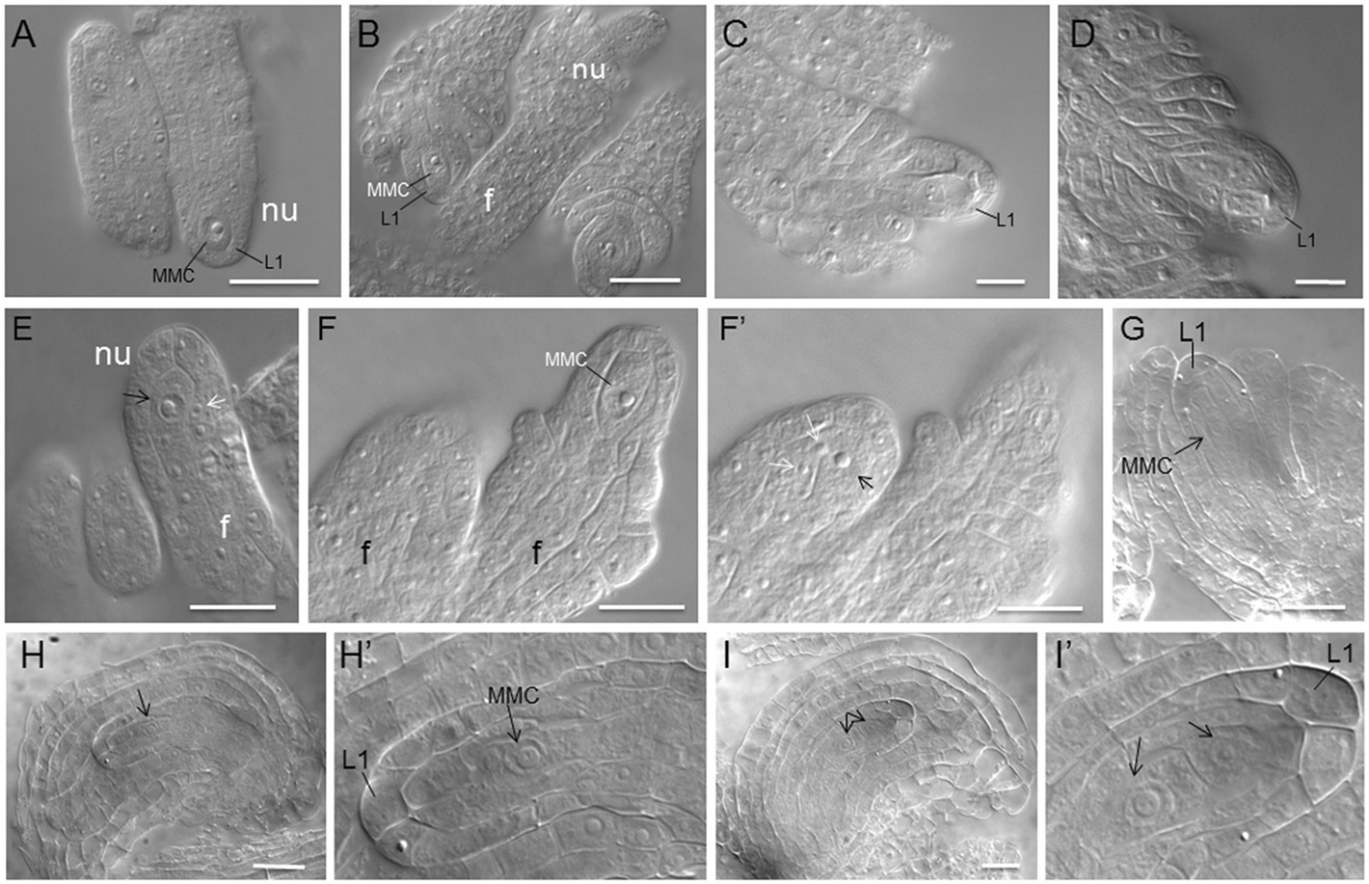
**Early ovule development is disrupted in 35S:NTAP:AtCTF7∆B plants.** Wild type **(A-D)** and 35S:NTAP:AtCTF7∆B **(E-I )** ovules were analyzed by differential interference contrast microscopy. **(A)** Pre-meiotic ovule containing a single megaspore mother cell (MMC; stage 1-II). **(B)** Pre-meiotic ovule at stage 2-III. Inner and outer integuments start to initiate. **(C)** Meiotic ovule containing a dyad after meiosis I (stage 2-IV). **(D)** Meiotic ovule containing a tetrad after meiosis II. **(E-F)** 35S:NTAP:AtCTF7∆B ovules at pre-meiotic stages. **(E)** Right ovule containing an abnormal, enlarged cell (white arrow) adjacent to a MMC-like cell (black arrow). Adjacent ovules are different in size. **(F-F’)** Left ovule containing two abnormal, enlarged cells (white arrows) adjacent to a MMC-like cell (black arrow). Ovules are different in size and stage (left: stage 1-II, right: stage 2-III) and point in the same direction. **(G-I)** 35S:NTAP:AtCTF7∆B ovules at meiosis. **(G)** Ovule containing a large cell with prominent nucleus (arrow) resembling a MMC. An extra cell is present at the position of the degenerated megaspores, between the MMC and L1 cells. **(H)** Ovule containing a MMC-like cell with a prominent nucleus in the central region of the ovule. Ovule is enlarged and extra cells are between the MMC and L1 cells. **(H’)** Magnified view of MMC from **H**. **(I)** Ovule containing two cells with prominent nuclei (arrows) in the central region of the ovule. The two cells are separated and resemble a dyad. Extra cells surround the dyad. **(I’)** Magnified view of **I**. Size bar = 10 μm. Developmental stages are defined according to Schneitz et al. [32].

To elucidate the function(s) of 35S:NTAP:AtCTF7∆B in ovule development, ovules from wild type and Line 11 plants were analyzed by differential interference contrast (DIC) microscopy. In wild type siliques, archesporial cells are specified from the subepidermal cell layers and differentiate into megaspore mother cells, which are initially unpolarized and become polarized prior to the start of meiosis (Additional file 1: Figure S3A). Two rounds of meiosis produce a tetrad of four haploid megaspores (Figure 3C,D; Additional file 1: Figure S3B-E) [32]. Prior to FG1, the megaspore mother cell, dyad and tetrad are all adjacent to L1 cells (Figures 3A-D) [33]. When the ovule reaches FG1, the megaspore at the chalazal-end differentiates into the functional megaspore while the other three megaspores undergo programmed cell death (Additional file 1: Figures S3F, G; Figures S4B, C) [34,35]. In wild type siliques, adjacent ovules are similar in size and point in opposite directions (Figures 3A,B).

In mutant plants*,* defects were observed in ovules very early in development. Approximately 30.4% (41/135) of the ovules observed were defective at the premeiosis stage. Abnormally enlarged sub-epidermal cells were observed adjacent to normal looking megaspore mother cells in some pre-meiotic ovules (Figure 3E,F’). Adjacent ovules were often different in size and sometimes pointed in the same direction (Figure 3 F,F’). A functional megaspore was not identified in some ovules even though their shape and size was beyond FG1. Instead, large cells with prominent nuclei and megaspore-like characteristics [36] were found in the position of a normal embryo sac (Figures 3G-I). Ovules containing either one megaspore-like cell (Figure 3G,H) or two megaspore-like cells with dyad characteristics (Figure 3I) were also observed. This suggested that meiosis is delayed, altered and arrested in most megaspores. Extra cells were also observed between the megaspores and the L1 layer of cells (Figure 3G-I). In WT plants, a small number (6.7%, 8/120) of ovules were found to contain twin megaspore mother cells; however no defects were observed during or after meiosis. In Line 11 plants, 14.1% (19/135) of the ovules contained multiple megaspore mother-like cells at pre-meiosis. Furthermore, 72.5% (50/69) of ovules with dyads contained extra cells between the dyad and L1 layer, and 27.5% (19/69) the dyads were in elongated shape. Ultimately 50.4% (64/127) of the ovules observed were defective at FG1 stage. Therefore, alterations in archesporial cell differentiation, the onset and progression of meiosis and possibly somatic cell identity are observed in ovules of 35S:NTAP:AtCTF7∆B plants.

**Figure 4:**
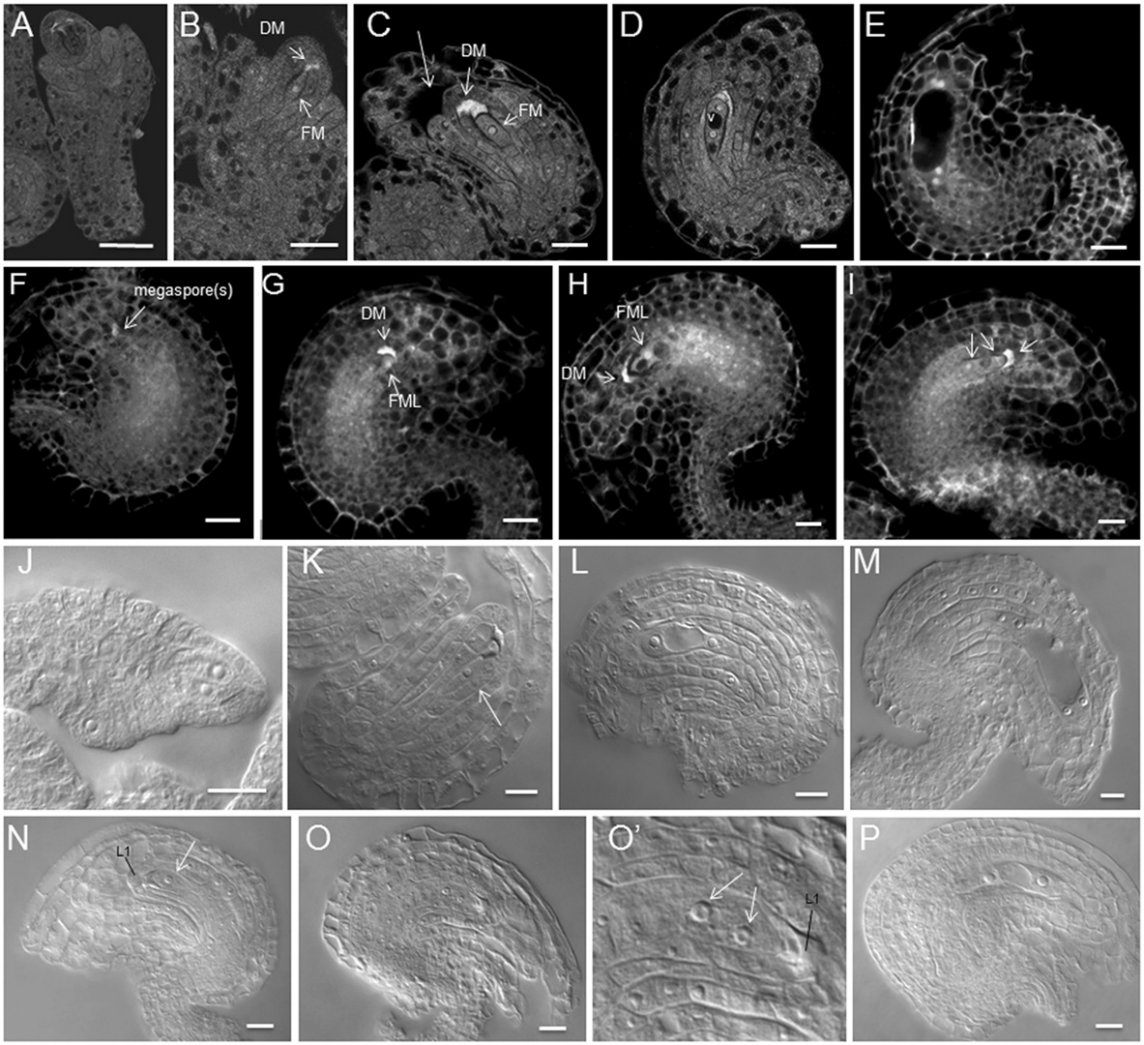
**Embryo sac development is delayed and arrests early in 35S:NTAP:AtCTF7∆B ovules. (A-E)** and **(J-M)** Wild type ovules. **(F-I)** and **(N-P)** 35S:NTAP:AtCTF7∆B ovules. **(A)** FG0 ovule. No FM is identified. **(B)** Early FG1 ovule showing the FM and DM. The nucellus is not surrounded by the integument. **(C)** FG1 ovule. The nucellus is surrounded by the outer integument but not the inner integument. **(D)** FG3 ovule containing a two-nucleate embryo sac. The nucellus is enclosed by the inner integument. **(E)** FG4 ovule containing a four-nucleate embryo sac. **(F-I)** Embryo sac development in 35S:NTAP:AtCTF7∆B ovules observed by CLSM. **(F)** Ovule containing megaspore(s). **(G)** Ovule containing a FG1 embryo sac. Chalazal end megaspore becomes functional megaspore like (FML) and the other megaspores are degrading. **(H)** FG1 embryo sac. FML locates at a more chalazal position. **(I)** FG3 embryo sac containing two nuclei with a vacuole between them. **(J-M)** Embryo sac development in WT ovules visualized by DIC (also see Additional file 1: Figure S3). **(J)** Meiotic ovule containing a dyad (stage 2-IV). **(K)** FG1 ovule. FM (arrow) is uni-nucleate. **(L)** FG3 ovule, containing an embryo sac with two nuclei and a vacuole. **(M)** FG4 ovule. **(N-P)** Embryo sac development in 35S:NTAP:AtCTF7∆B ovules observed by DIC. **(N)** FG1 embryo sac. The nucellus is surrounded by the outer integument and the inner integument. Extra cells are present between the FM (arrow) and L1 cells. **(O-O’)** Ovule containing a two-nucleate embryo sac. Non-degenerated L1 cells are present. **(P)** Ovule with a FG3 embryo sac. FMLs are identified as having distinctly bright nuclear autofluorescence and DMs contain a diffuse signal throughout the cells, but no clearly defined nucleus, defined according to Barrell and Grossniklaus [37]. Size bar = 10 μm. Developmental stages in CLSM and DIC are defined according to Christensen et al. [34,35] and Schneitz et al. [32], respectively.

The effects of 35S:NTAP:AtCTF7∆B on megagametogenesis were investigated by confocal laser scanning microscopy (CLSM) and DIC microscopy. During wild type megagametogenesis, the functional megaspore undergoes three rounds of mitosis accompanied by nuclear migration, fusion, degeneration and cellularization to form the final embryo sac (Additional file 1: Figures S3H-L; S4D-J). At FG1, the functional megaspore undergoes mitosis to produce a two-nucleate embryo sac (FG2; Additional file 1: Figure S3H). Formation of a vacuole between the two nuclei marks stage FG3 (Figure 4,D and L; Additional file 1: Figures S3I, S4D). During FG3 the ovule becomes curved and the inner integument embraces the nucellus. A second round of mitosis produces a four-nucleate embryo sac (FG4; Figure 4E,M; Additional file 1: Figures S3J, S4E). This is followed by migration of the two chalazal nuclei from an orthogonal orientation to a chalazal-micropylar orientation. After nuclear migration, a third round of mitosis gives rise to eight nuclei in a 4n + 4n configuration (FG5; Additional file 1: Figure S3K). The two polar nuclei, one from each side, meet at the embryo sac’s micropylar half and fuse to form the central cell, while the antipodal nuclei start to degenerate (Additional file 1: Figure S4H). The central cell has formed and the antipodal nuclei are completed degenerated by FG7 (Additional file 1: Figures S3K, S4I). Prior to fertilization, one synergid nucleus degenerates, such that the embryo sac consists of one egg cell, one central cell and one synergid nucleus (FG8; Additional file 1: Figures S3L, S4J).

Ovule development is typically synchronous in wild type sliques with predominately one or two developmental stages present in a given pistil (Additional file 1: Table S2). In contrast, 35S:NTAP:AtCTF7∆B ovule development appeared slowed and asynchronous (Additional file 1: Table S3). Female gametophytes in the same pistil were often at several different stages, indicating that the synchrony of gametophyte development was disturbed and embryo sac maturation was delayed. Ovules containing a megaspore (Figures 3G,H’), FG1 embryo sacs with a functional megaspore-like cell [37] and degrading megaspores (Figures 4G,H,N), and abnormal FG2 and FG3 embryo sacs were commonly observed in the same slique (Figure 4I,O-P). Ovules containing degraded/degrading megasporocytes, and degrading FG1 embryo sacs were also observed (Additional file 1: Figures S5A-D). Alterations in nuclear division appeared to precede arrest in some megaspores (Additional file 1: Figure S5E-H). Most embryo sacs arrest at FG2/FG3; although some terminal ovules appeared to progress beyond FG3 (Additional file 1: Table S3). Common phenotypes included degraded/degrading nuclei (Additional file 1: Figure S5J-K), degenerated embryo sacs (Additional file 1: Figure S5M), polar nuclei fusion defects (Additional file 1: Figure S5N) and vacuole development defects (Additional file 1: Figure S5N-P).

**Figure 5:**
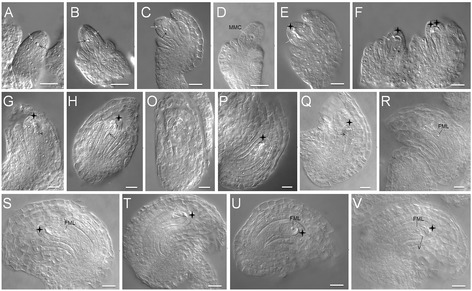
**Phenotypes of 35S:NTAP:AtCTF7∆B ovules from Line 15. (A-C)** Pre-meiotic 35S:NTAP:AtCTF7∆B ovules containing abnormal enlarged cells (white arrows) adjacent to megaspore mother cell-like cells (MMC like; black arrows). **(D)** Pre-meiotic ovule containing a MMC with degenerating nucleus. **(E-Q)** Meiotic ovules containing abnormal enlarged cells (white arrows) adjacent to DMs (stars). Cells with functional megaspore-like (FML) characteristics are indicated by black arrows in **F**, **G**, **H** and **Q**. The abnormal cell in Q contains two nuclei (white arrows). **(R**-**S)** Post-meiotic ovules containing FMLs. **(R)** The FML is associated with abnormal cells, which are not degenerating (white arrows). **(S)** Extra cells/nuclei are present between the DM (star) and FML. **(T)** Ovule containing two abnormal cells (white arrows) adjacent to the DM (star). **(U-V)** Post-meiotic ovules containing female gametophytes with one nucleus. DM, degenerated megaspore are denoted by stars; FML, functional megaspore like; MMC, megaspore mother cell. Size bars, 10 μm.

To determine if the alterations observed in Line 11 are representative of 35S:NTAP:AtCTF7∆B plants in general, ovules from other lines exhibiting severely reduced fertility (#13 and #15) were examined. Similar to the situation in Line11, pre-meiotic ovules from Line15 contained abnormally enlarged sub-epidermal cells adjacent to megaspore mother-like cells (Figures 5A-C). During meiosis, ovules contained abnormal cells adjacent to degenerated megaspores (Figures 5D-Q). Some ovules contained functional megaspore-like cells (Figure 5F-H,Q). Mature ovules contained differentiated functional megaspore-like cells (Figures 5R,S) and female gametophytes with various defects (Figures 5U,V). Similar to Line 11, female gametophytes from Lines 13 and 15 developed slowly and asynchronously; embryo sacs arrested at FG1 and FG3 (Additional file 1: Figure S6A,C). Additional alterations not observed in Line 11 were also identified, including some ovules that appeared to contain two functional megaspore-like cells (Additional file 1: Figure S6B). In some ovules the middle megaspores appeared to differentiate into functional megaspore-like cells while the megaspores at the chalazal-end degenerated (Additional file 1: Figure S6D), suggesting that ovule polarity was disrupted. All together, 30.4% (48/158) ovules examined in Line 15 plants displayed alterations at pre-meiosis; 66.0% (68/103) of the ovules were defective at meiosis and 79.3% (73/92) of the ovules were defective at FG1. Therefore, common defects associated with NTAP:AtCTF7∆B include a delay and alterations in both megasporogenesis and megagametogenesis with embryo sacs arresting at various stages of development.

Because the ovules of 35S:NTAP:AtCTF7∆B plants appeared to exhibit a delay in the onset of meiosis, qRT-PCR was carried out to examine transcript levels for several genes important for meiosis and ovule development (Additional file 1: Figure S7A,B). Transcripts for *WUS1* [38], *MMD1* [39], *SPO11-1* [40] and *ZYP1a* [41] were elevated between two and three fold in 35S:NTAP:AtCTF7∆B plants relative to wild type. Transcript levels of *DMC1* [42], *SYN3* [43,44] and *OSD1* [45] showed modest increases, while the transcript levels of other genes were unchanged (Additional file 1: Figures S7A,B).

### Expression of 35S:NTAP:AtCTF7∆B causes pleiotropic growth defects

**Figure 6:**
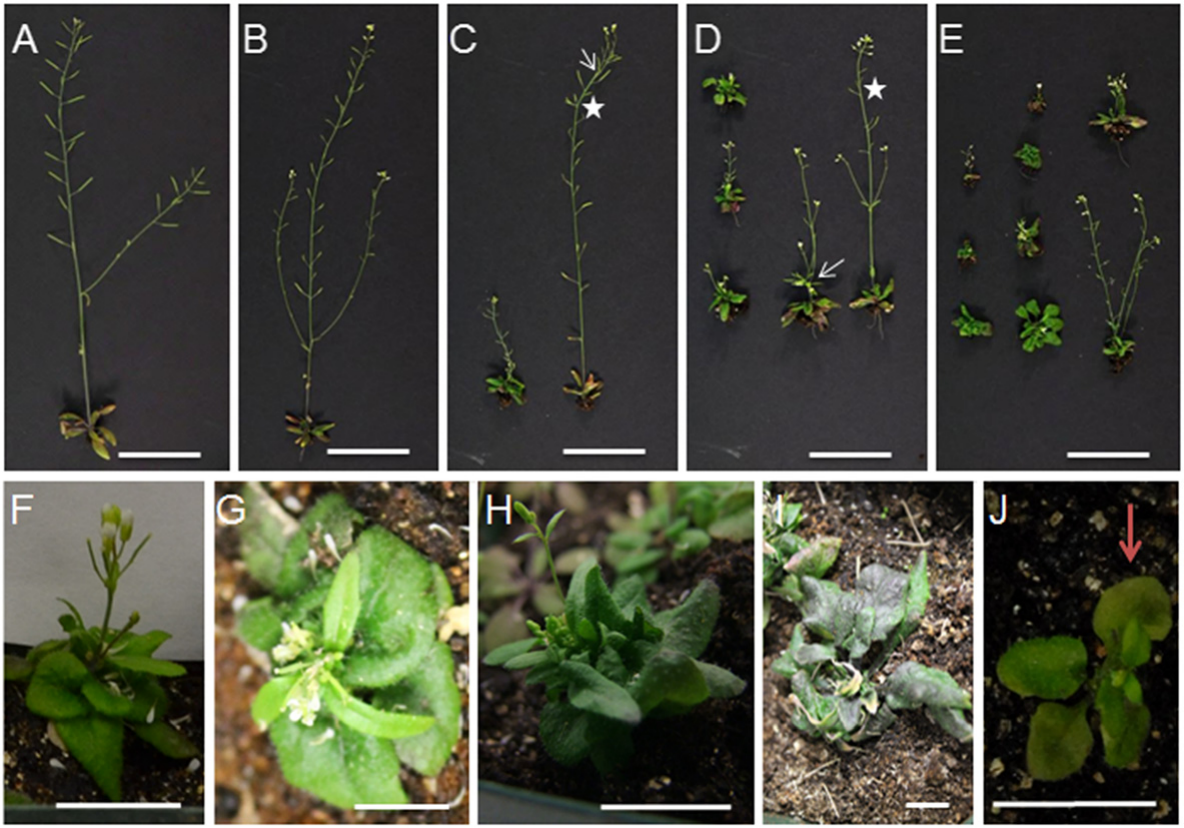
**35S:NTAP:AtCTF7∆B plant morphology changes in later generations. (A)** Wild type Columbia plant. **(B-E)** Morphological alterations get progressively worse in 35S:NTAP:AtCTF7∆B plants through self-pollination. **(B)** Second generation plants are normal, but reduced fertile. **(C)** Both dwarf and non-dwarf, reduced fertile plants are observed in third generation plants. **(D)** Fourth generation plants. **(E)** Sixth generation plants, showing a higher frequency of dwarf plants. Defects such as reduced apical dominance (arrow) and phyllotaxis disturbances (asterisks) are observed. **(F-I)** Representative 35S:NTAP:AtCTF7∆B dwarf plants. Inflorescence defects include acaulescent **(G)**, multiple inflorescence branches at the first node **(H)** and no inflorescence **(I)**. Leave defects include aberrant rosette size and shape **(F, H and I)**. **(J)** *atctf7-1* plant showing an early senescence phenotype (arrow). Plants **B** to **I** are from Line 11. All plants are grown under the same conditions. Plants in **A**-**H** and **J** are approximately 30 days old; plant in I is 40 days old. Scale bar = 5 cm in **A**-**E** and 2 cm in **F**-**J**.

During the analysis of 35S:NTAP:AtCTF7∆B reduced fertility lines, plants displaying vegetative defects began to appear in the T2 or T3 generations. Specifically, later generations grew progressively worse in 12 of the 18 independent severely reduced fertility lines examined. The remaining six lines continued to display reduced fertility, but did not exhibit vegetative defects through the seventh generation*.* A wide range of morphological defects was observed in the 12 lines (Figure 6). The defects varied between lines and between progeny of the same line. The observed vegetative abnormalities included dwarf plants, fused stems and disruption of phyllotaxis (Figures 6C-E).

The proportion of plants exhibiting vegetative alterations increased in successive generations. Likewise, the severity of the vegetative alterations also became successively worse in subsequent generations. For example, the frequency of dwarf plants increased from approximately 18% in the third generation to 82% by generation six (Figure 6; Table 1). The dwarf phenotype also became more severe with later generations containing smaller, more defective plants. Dwarf plants varied in morphology and exhibited a range of alterations, including acaulescent plants, floral abnormalities, homeotic changes and irregular leaves (Figure 6F-I). While some of the most severe 35S:NTAP:AtCTF7∆B dwarf plants resembled *atctf7-1* plants (Figure 6J), in general the phenotype was less severe than *atctf7-1* plants. Furthermore, while most *atctf7-1* plants exhibit early senescence, 35S:NTAP:AtCTF7∆B plants typically did not. In order to determine if the phenotypic alterations were due to increased 35S:NTAP:AtCTF7∆B expression, native CTF7 and total CTF7 levels were measured in both dwarf and non-dwarf plants of three different generations of 35S:NTAP:AtCTF7∆B plants, which showed progressively more severe phenotypes. Native CTF7 levels ranged from 2.6-3.7 fold above wild type in the different plants, while total CTF7 transcript levels were elevated 5.4-7.6 fold relative to wild type. However, no consistent difference was observed between dwarf and non-dwarf plants or between one generation and the other. Therefore, the phenotypic differences observed are not due to dramatic changes in AtCTF7∆B transcript levels.

**Table 1:** **Non-Mendelian inheritance in 35S:NTAP:AtCTF7∆B plants**

**Generation**	**Dwarf plants (%)**	**Reduced fertile, non-dwarf plants (%)**
2nd		125 (100%)
3rd	24 (18.5%)	106 (81.5%)
4th		
Seeds from dwarf Parent	16 (42.1% )	22 (57.9%)
Seeds from non-dwarf Parent	25 (45.5%)	30 (54.5%)
5th		
Seeds from dwarf plants	55 (78.6%)	15 (21.4%)
Seeds from non-dwarf plants	81 (67.5%)	39 (32.5%)
6th		
Seeds from dwarf plants	55 (81.4%)	13 (18.6%)
Seeds from non-dwarf plants	66 (82.5%)	14 (17.5%)

Plants were from Line 11. Seeds from reduced fertile, non-dwarf plants and from dwarf plants were collected and sown separately.

**Figure 7:**
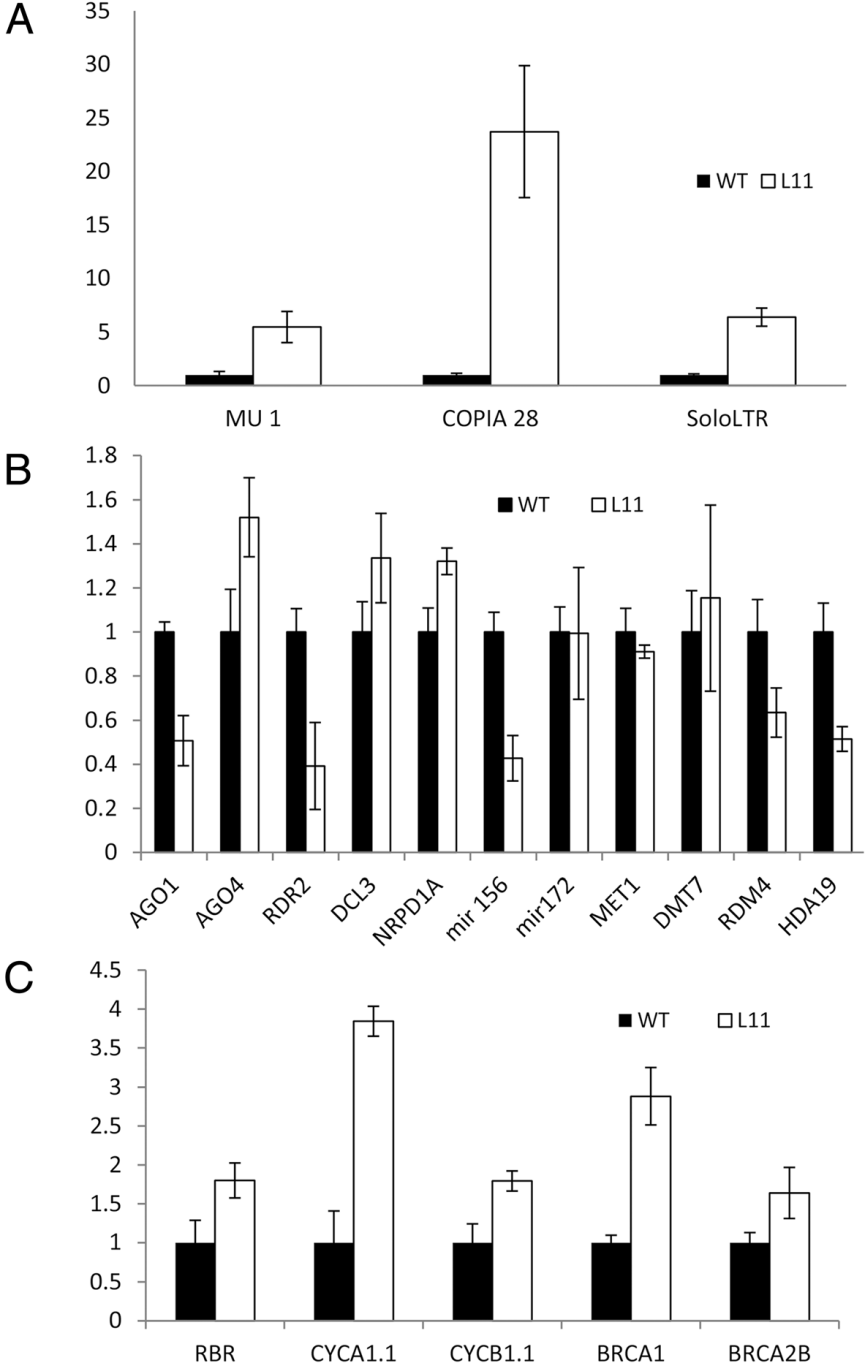
**Transcript levels of epigenetically regulated transposable elements and other select genes in 35S:NTAP:AtCTF7∆B plants. (A)** Transcript levels of *MU1*, *COPIA28* and *soloLTR*, are increased dramatically in 35S:NTAP:AtCTF7∆B plants. **(B)** Transcript levels of genes associated with epigenetic events are differently affected. *HDA19* and *RDM4* transcript levels are decreased, while *MET1* and *DMT7* transcripts are not altered. **(C)** Transcript levels of cell cycle genes, *CYCB1.1*, *CYCA1.1* and *RBR*, and DNA repair genes, *BRCA1* and *BRCA2B*, are increased. Buds of wild type and non-dwarf, reduced fertile 4th generation Line 11 plants were used. Data are shown as means ± SD (*n *= 3).

Rather, and most surprisingly, the dwarf phenotype was not inherited in a Mendelian fashion (Table 1). When dwarf plants were selfed they produced a mixture of dwarf and non-dwarf, reduced fertile plants. The frequency of dwarf plants produced from selfed dwarf plants was similar to the frequency of dwarf plants resulting from selfing a non-dwarf plant. The stochastic appearance of the dwarf phenotype and variation in phenotypes suggested that the alterations could be the result of epigenetic changes. In order to investigate this possibility, qRT-PCR was carried out to measure the expression levels of several epigenetically regulated transposable elements (TEs), including *MU1*, *COPIA 28* and *solo LTR* [46], as well as several genes associated with epigenetic events [47-50]. Expression levels of *MU1*, *COPIA 28* and *solo LTR* were increased between five (*MU1*) and 24 fold (*COPIA 28*) in 35S:NTAP:AtCTF7∆B plants (Figure 7A). Subtle changes were also observed in the transcript levels of several siRNA associated genes (Figure 7B). *ARGONAUTE1* (*AGO1), RDR2* and *mir156* transcript levels were reduced approximately 40-60% while *AGO4* transcripts were elevated slightly. Transcript levels of *HDA19* and *RDM4* were also decreased approximately 50% (Figure 7B), while transcript levels of the canonical DNA methylation genes, *MET1* and *DMT7*, did not vary significantly (Figure 7B).

Transcript levels of several cell cycle (*RBR, CYCB1.1* and *CYCA1.1*) and DNA repair (*BRCA1* and *BRCA2B*) genes have previously been shown to be elevated in *atctf7* plants [17]. These genes were tested and found to also be elevated in 35S:NTAP:AtCTF7∆B plants (Figure 7C). The similarities in morphological defects and expression patterns observed between 35S:NTAP:AtCTF7∆B and *atctf7-1* plants [17] are consistent with the hypothesis that the 35S:NTAP:AtCTF7∆B construct is exerting a dominant negative effect.

Finally, experiments were carried out to determine if the 35S:NTAP:AtCTF7∆B-associated defects are due to the presence of the N-terminal tag or the absence of the acetyltransferase B motif. A 35S:AtCTF7∆B construct was generated and transformed into wild type Columbia plants (Additional file 1: Figure S1). Eight out of the 13 35S:AtCTF7∆B transgenic lines examined exhibited reduced fertility, with the 35S:AtCTF7∆B defects typically appearing more severe than those in 35S:NTAP:AtCTF7∆B plants (Additional file 1: Figure S8). Like 35S:NTAP:AtCTF7∆B plants, dwarf plants were not observed until the third generation in 35S: AtCTF7∆B plants. However, dwarf plants appeared at higher frequencies and their phenotypes were more varied than 35S:NTAP:AtCTF7∆B plants (Additional file 1: Figure S8B). For example, the viability of Line 21 decreased with successive generations, such that seeds from this line were not viable by the 4th generation. Likewise, while abnormalities associated with 35S:AtCTF7∆B plants were similar to those observed in 35S:NTAP:AtCTF7∆B plants, additional alterations were also present. For example, 35S:AtCTF7∆B Line 24 segregated for two types of plants, those without an inflorescence and “normal” reduced fertility plants (Additional file 1: Figure S8C). 35S:AtCTF7∆B Line 29 plants produced siliques that pointed downward and contained fewer seeds (46.4 ± 2.5 versus 54.2 ± 4.1 per silique in WT; n = 35) (Additional file 1: Figure S8D). This phenotype is similar to *bp/knat1* mutations [51].

Finally, in order to investigate which aspect(s) of the 35S:NTAP:AtCTF7∆B construct was causing the fertility and growth defects, several additional constructs were generated and introduced into wild type or *Atctf7-1*^+/−^ plants. Wild type plants transformed with a 35S:NTAP:AtCTF7 construct (Additional file 1: Figure S1) resembled 35S:AtCTF7 plants [16]. Specifically, the plants grew normally and produced pollen, but exhibited reduced female fertility. A CTF7:AtCTF7∆B construct (Additional file 1: Figure S1) was also created and transformed into *Atctf7-1*^+/–^plants. The native CTF7 promoter is expressed at low levels throughout the plant [17]. Wild type plants containing the CTF7:AtCTF7∆B construct exhibited normal growth and development and normal fertility levels (52.0 ± 2.2 seed/silique, n = 33 versus 54.2 ± 4.1 seed/silique in wild type, n = 35). No alterations were observed in six different transgenic lines over six generations. *atctf7-1* plants containing the CTF7:AtCTF7∆B construct were obtained in T2 populations, but at frequencies (7%, 6/86) much lower than expected (25%) if the construct complemented the *atctf7-1* mutation. While these plants were dwarf, they grew better than *atctf7-1* plants (Additional file 1: Figure S8E). Somewhat similar to 35S:AtCTF7∆B plants, plant morphology varied between plants with some plants appearing acaulescence or producing fewer rosette leaves. The plants produced approximately 40 ovules per silique; however they failed to set seed; siliques contained aborted ovules resembling *atctf7-1* plants (Additional file 1: Figure S8G). Therefore, the defects observed in 35S:NTAP:AtCTF7∆B plants are caused by high-level expression of AtCTF7∆B and not the presence of the NTAP tag. Rather, the presence of the NTAP appears to reduce the severity of the alterations, either by reducing the stability or activity of the protein, possibly by affecting its interaction with other proteins. Further, expression of a truncated version of CTF7, missing the B motif, can restore some vegetative growth to *atctf7-1* plants; however the plants are still completely sterile. Therefore, an intact acetyltransferase domain is required for full CTF7 activity.

## Discussion

The acetylation of cohesin complexes at conserved lysine residues by CTF7/Eco1 plays an essential role in the establishment of cohesion during S phase and therefore nuclear division. Consistent with this, CTF7/Eco1 null mutations are typically lethal. Organisms expressing altered CTF7/Eco1 levels or point mutations in the protein typically display relatively normal levels of cohesin during nuclear division, but exhibit a range of developmental alterations. For example, mutations in the N-terminus of the protein typically lead to defects in cohesion and often chromosome loss during mitosis [52]. In contrast certain mutations in the C-terminal acetyltransferase domain of yeast CTF7/ECO1 have little effect on S-phase cohesion and chromosome segregation, but cause an increased sensitivity to DNA-damaging agents. Likewise, Roberts Syndrome in humans has been linked to point mutations in ESCO2. Cells from patients with Roberts Syndrome are typically hypersensitive to DNA-damaging agents and show premature centromere separation; however, only 10–20% of cells show abnormal mitosis [27,53]. Finally, numerous studies have shown that cohesin mutations or reductions in cohesin levels result in transcriptional alterations that can have far-ranging developmental consequences [54].

Generally similar results have been obtained from studies on CTF7 in plants. Arabidopsis plants heterozygous for a T-DNA insertion in *AtCTF7* grow normally but produce approximately 25% aborted seeds, consistent with the conclusion that CTF7 is an essential protein [16]. Likewise knockdown of *AtCTF7* mRNA levels leads to growth retardation and defective sister chromatid cohesion [18]. However, unlike other organisms, homozygous *Atctf7* plants have been detected at very low frequencies [17]. *Atctf7* plants exhibit a wide range of developmental defects, including extreme dwarfism and sterility.

In our current study we show that high-level expression of a truncated form of AtCTF7 results in reduced fertility and dramatic alterations in vegetative growth. Specifically, plants that express a 35S:NTAP:AtCTF7_∆299–345_ construct exhibit defects in male and female meiocytes, with female reproduction being affected more dramatically. Male meiocytes exhibited chromosome fragmentation and uneven chromosome segregation during meiosis II that resulted in abnormal pollen development and ultimately pollen abortion. Ovules contained abnormal megasporocyte-like cells during pre-meiosis, megaspores that experienced elongated and aborted meiosis and defective megaspores and embryo sacs that arrested at various stages. A broad range of vegetative defects was also observed beginning in T_2_ generations of AtCTF7∆B transgenic plants. The appearance of these defects was stochastic and inherited in a non-Mendelian fashion. Comparison of AtCTF7∆B transgenic plants with *AtCTF7* RNAi and *atctf7* plants and CTF7/Eco1 mutants in other organisms suggests that CTF7 may have multiple roles in the cell.

### Reproductive defects in 35S:NTAP:AtCTF7∆B plants differ from those in AtCTF7RNAi and *atctf7* plants

Inactivation of *AtCTF7* by T-DNA insertion or a reduction in *AtCTF7* levels by *AtCTF7*-RNAi lead to alterations in chromosome condensation and sister chromatid cohesion during early meiotic prophase followed by defects in homologous chromosome pairing and segregation later in meiosis [17,18]. The effect of 35S:NTAP:AtCTF7∆B on male meiocytes was less severe and occurred later in meiosis. The first noticeable defect was the appearance of lagging and broken chromosomes during telophase I (Figure 2H). Twenty or more individual chromosomes were often observed beginning at metaphase II, suggesting that cohesion might be prematurely lost. Overexpression of AtCTF7∆B did not have a noticeable effect on the initial establishment of cohesion, as the distribution of SYN1 on meiotic chromosomes was normal throughout prophase (Additional file 1: Figure S2). Likewise, chromosome condensation, sister chromatid cohesion and homologous chromosome pairing were normal during male meiotic prophase in 35S:NTAP:AtCTF7∆B plants. This suggests that AtCTF7∆B does not affect the bulk of meiotic cohesin complexes to a significant extent, but rather may alter centromeric cohesin levels, or possibly cohesin interactions with SGO1 or PATRONUS [55].

In contrast to male reproduction, the effect(s) of 35S:NTAP:AtCTF7∆B on female reproduction are observed earlier and are more variable than those in *atctf7* and *AtCTF7* RNAi plants. Over expression of *AtCTF7* from the 35S promoter or knockdown of *AtCTF7* using RNAi blocks early ovule development, typically at FG1 or FG2 [16,18]. In contrast, *atctf7* ovules in AtCTF7^+/–^plants develop normally, but arrest soon after fertilization [16]. In all three situations the alterations are relatively uniform with arrest occurring at a specific developmental stage. In contrast, 35S:NTAP:AtCTF7∆B causes pleiotropic ovule/seed defects. NTAP:AtCTF7∆B lines displayed a wide range of defects, including additional abnormal cells adjacent to gametic cells, delayed/arrested meiosis, the production of functional megaspore-like cells of which some are mis-positioned, and delayed and altered embryo sac development. Although alterations were commonly first observed prior to and during meiosis, most megaspores progressed to FG2 or FG3 before arresting (Additional file 1: Table S3).

### 35S:NTAP:AtCTF7∆B leads to defects consistent with epigenetic alterations

35S:NTAP:AtCTF7∆B lines exhibited relatively normal vegetative growth and development for the first two generations. However, severe vegetative abnormalities began to appear starting in the T2 or T3 generations of different lines. The defects, which included dwarf plants, fused stems and disrupted phyllotaxis (Figure 6C-E), varied between lines and between progeny of the same line. The proportion of plants exhibiting vegetative alterations as well as the severity of the vegetative alterations increased in successive generations. It is interesting to note that phenotypic variability is very common in RBS patients [26,27].

The delayed appearance of vegetative defects and the increased frequency of defects in subsequent generations could result from the accumulation of defects in 35S:NTAP:AtCTF7∆B plants. Consistent with this possibility is the observation that *ctf7/eco1* mutations are commonly associated with sensitivity to DNA damaging agents [56]. As expected, both 35S:NTAP:AtCTF7∆B and *atctf7* plants contain elevated transcript levels for DNA repair and recombination genes (Figure 7; Additional file 1:Figure S7) [17]. While the vegetative defects in 35S:NTAP:AtCTF7 plants may result from spontaneous mutations, the situation is clearly more complex as the dwarf phenotype is not inherited in a Mendelian fashion (Table 1). When dwarf plants were selfed they produced a mixture of dwarf and non-dwarf, reduced fertile plants. Further, the frequency of dwarfs in the progeny of selfed dwarf plants was similar to the frequency of dwarf plants resulting from selfing of non-dwarf plants. This raised the possibility that epigenetic alterations may be present in 35S:NTAP:AtCTF7∆B plants. Consistent with this possibility is our observation that transcript levels of *MU1*, *COPIA 28* and *solo LTR* were increased between five (*MU1*) and 24 fold (*COPIA 28*) in 35S:NTAP:AtCTF7∆B plants (Figure 7A). Subtle changes were observed in the transcript levels of several siRNA associated genes. *AGO1*, *RDR2* and *mir156* transcript levels were reduced approximately 40-60% while *AGO4* was elevated slightly. *HDA19* and *RDM4* transcripts were also decreased approximately 50% (Figure 7B).

Our observation that epigenetic alterations may be present in 35S:NTAP:AtCTF7∆B plants is consistent with the alterations we observe in female reproduction. Recent studies have shown that embryo sacs are enriched for transcripts of proteins involved in RNA metabolism and transcriptional regulation, and that they display distinct epigenetic regulatory mechanisms [57-59]. Disruption of genes in small RNA regulatory pathways, such as *AGO1, AGO9, DICER-LIKE1 (DCL1)* and *MEIOSIS ARRESTED AT LEPTOTENE1 (MEL1),* leads to multiple gametic cells at premeiosis, abnormal meiotic divisions, gametic cell fate alterations and twin female gametophytes [33,59,60]. For example, mutations in *AGO9*, which participates in small RNA silencing by cleaving endogenous mRNAs, results in additional gametic cells in pre-meiotic ovules, which may skip meiosis and twin female gametophytes in post-meiotic ovules [33]. Several of these defects are observed in 35S:NTAP:AtCTF7∆B ovules before and during meiosis. Moreover, AGO9 participates in the epigenetically regulated silencing of TEs [33]. At this time it is not clear if the apparent epigenetic alterations we observed are the direct result of high-level NTAP:AtCTF7∆B expression or a secondary effect. For example, it is possible that NTAP:AtCTF7∆B expression directly affects the expression of genes involved in epigenetic regulation. The involvement of cohesin complexes in transcriptional regulation is well documented in other organisms [54]. It is also possible that the changes we observe are an indirect effect of NTAP:AtCTF7∆B expression. For example, previous studies have shown that *eco1/ctf7* mutations result in defects in nucleolar integrity, rRNA production, ribosome biogenesis and protein biosynthesis in *Saccharomyces cerevisiae* and human [25,61]. In Arabidopsis, mutations in genes participating in mRNA production and rRNA/ribosome biogenesis slow mitotic progression in female gametophytes and result in pleiotropic defects in embryo sacs [58,59,62-66]. For example, mutations in *SLOW-WALKER1 (SWA1*), which participates in 18S pre-rRNA processing, results in asynchronous megagametophyte development, and embryo sac arrest over a wide range of stages [62]. Likewise, mutations in ribosomal protein genes lead to defects in inflorescence, leaf and plant stature in Arabidopsis, similar to those observed in 35S:NTAP:AtCTF7∆B plants [66-69]. Therefore, many of the alterations we observe in 35S:NTAP:AtCTF7∆B plants could be the result of alterations in rRNA biogenesis or ribosome biogenesis, which in turn could indirectly impact epigenetic pathways.

### AtCTF7∆B likely acts on several levels

The alterations observed in 35S:NTAP:AtCTF7∆B plants appear to result from the presence of high levels of AtCTF7∆B and not a reduction of native AtCTF7 levels or the presence of the NTAP. 35S:AtCTF7∆B plants exhibit similar, if not more dramatic phenotypes than 35S:NTAP:AtCTF7∆B plants, indicating that the NTAP is not responsible for the observed phenotypes. Likewise, expression studies show that in addition to high *AtCTF7∆B* transcript levels, transgenic plants also contain elevated levels of native *AtCTF7* transcripts (Figure 1C). Therefore, the 35S:AtCTF7∆B construct does not cause co-suppression. Consistent with this are the apparently normal SYN1 cohesin patterns observed in male meiocytes (Additional file 1: Figure S2). Interestingly, the elevated levels of native *AtCTF7* transcript suggest that the cellular cohesion status is altered to some extent in AtCTF7∆B plants and that a feedback loop exists to monitor and maintain cohesion levels in plants.

High-level expression of AtCTF7∆B appears to exert a dominant negative effect, resulting in a relatively wide range of alterations. Less clear is how high level expression of AtCTF7∆B exerts its effect or if the alterations we observe are all related. Deletion of the last 46 amino acids of the acetyltransferase domain is expected to eliminate most of the actyltransferase activity. High-level expression of the protein may directly compete with native AtCTF7 for cohesin substrates resulting in an overall reduction or redistribution of cohesin levels throughout the genome. These changes could in turn result in a wide range of transcriptional alterations, similar to the situation observed in other organisms [54]. While this is the most-likely effect, it may not explain all of the observed alterations. For example, to our knowledge apparent epigenetic alterations have not been observed in either *AtCTF7* RNAi or *atctf7* plants [17,18]. Therefore, it is also possible that the 46 amino acid deletion alters acetyltransferase specificity such that the protein acts on off targets. For example, CTF7 has been shown to not acetylate histones; however if altered substrate specificity resulted in the acetylation of histones, then changes in chromatin structure could produce some of the alterations we observe. Finally, the possibility also exists that the deletion may alter the interaction of AtCTF7 with other proteins, either directly or indirectly involved in maintaining chromatin structure. Further experiments are required to determine how specifically AtCTF7∆B is acting, why male and female reproduction respond differently to alterations in AtCTF7 levels and what role, if any AtCTF7 plays in epigenetic regulation.

## Conclusions

Proper levels of AtCTF7 are critical for proper plant growth and development with female gametophytes being most sensitive to changes in AtCTF7 activity. High level expression of NTAP:AtCTF7∆B results in pleiotropic defects in reproduction and vegetative growth. High levels of AtCTF7∆B may affect small RNA processing, which in turn appears to result in epigenetic alterations. These results indicate that CTF7 may play multiple roles in plant cells.

## Methods

### Plant material and growth conditions

Wild type *Arabidopsis thaliana* plants (ecotype Columbia), the SALK_059500 (*ctf7-1*) insertion line and all transgenic plants described in this report were grown in Metro-Mix200 soil (Scotts-Sierra Horticultural Products; http://www.scotts.com) in a growth chamber at 22°C with a 16-h-light/8-h-dark cycle as described [16]. T-DNA insertion and transgenic plants were genotyped by PCR with primer pairs specific for the T-DNA and wild-type loci.

### Cloning procedures for construction of the transgenic plants

The AtCTF7∆B cDNA fragment (1–894 bp nucleotides, AtCTF7_∆299–345_) was digested with *Nde*I/*Hin*dIII and cloned into pIADL14 as described [16]. The 35S:NTAP:AtCTF7∆B construct was generated using Gateway-compatible binary vectors containing the NTAP tag ((NTAPi) [30]; a gift from Dr. Qinn Li lab, Miami Univeristy). AtCTF7∆B, 1–894 bp cDNA nucleotides, was first PCR-amplified with primers (1111/1201) and cloned into the pENTR vector, which was then fused with the binary vector by LR recombination reactions (Invitrogen) to make the final 35S:NTAP:AtCTF7∆B construct. 35S:AtCTF7∆B was generated by cloning the AtCTF7 cDNA (1–894 bp) as a *Nco*I/*Spe*I fragment into the binary vector pFGC5941. The 35S:NTAP:AtCTF7 construct was generated by the Gateway method with the corresponding primers. The AtCTF7 promoter (1.3 kb upstream of the ATG) was amplified, digested and cloned into the 35S:AtCTF7∆B construct. All the constructs were confirmed by DNA sequencing. The primers used are listed in Additional file 1: Table S4.

Each construct was mobilized into *Agrobacterium tumefaciens* strain AGL-1 and transformed into *Arabidopsis thaliana* using the floral dip method [70]. Transgenic plants were screened by BASTA and further confirmed by PCR.

### Quantitative real-time RT–PCR (qRT-PCR)

Buds of wild type and 35S:NTAP:AtCTF7∆B Line 11 4th generation plants were harvested and pooled separately. For 35S:NTAP:AtCTF7∆B samples, buds were only harvested from reduced fertile, non-dwarf plants or dwarf planys separately. Total RNA was extracted with the Plant RNeasy Mini kit (Qiagen, Hilden, Germany), and 10 μg of RNA was treated with Turbo DNase I (Ambion, http://www.invitrogen.com/site/us/en/home/brands/ambion.html) and used for cDNA synthesis with an oligo(dT) primer and a First Strand cDNA Synthesis Kit (Roche, http://www.roche.com). PCR was performed with the SYBR-Green PCR Mastermix (Bio-Rad, Hercules, CA, USA) and amplification was monitored on a MJR Opticon Continuous Fluorescence Detection System (Bio-Rad). Expression was normalized against β-tubulin-2. At least three biological replicates were performed, with two technical replicates for each sample. Student’s *t*-test was conducted to identify transcripts that exhibit statistically significant variation at the 95% confidence level. Sequences of primers used in these studies are presented in Additional file 1: Table S4.

### Chromosome analysis

Pollen morphology and viability were compared in flowers of 35S:NTAP:AtCTF7∆B plants and wild-type plants using Alexander staining [71]. Male meiotic chromosome spreads were carried out on floral buds fixed in Carnoy’s fixative (ethanol:chloroform:acetic acid: 6:3:1) and prepared as described previously [72]. Chromosomes were stained with 4,6-diamino-2-phenylindole dihydrochloride (DAPI, 1.5 μg ml^−1^; Vector Laboratories, Inc. Burlingame, CA, USA) and observed with an Olympus BX51 epifluorescence microscope system. Images were captured with a Spot camera system (Diagnostic Instruments Inc., http://www.spotimaging.com) and processed. Meiotic stages were assigned based on chromosome structure and morphology [72].

### Immunolocalization

SYN1 immunolocalization studies were carried out as previously described [31]. Primary antibodies for SYN1 were raised from rabbit and diluted 1:500. The slides were detected with Alexa Fluor 488 labeled goat anti-rabbit secondary antibody (1:2000; Molecular Probes, http://zt.invitrogen.com/). Slides were stained with DAPI and observed under an epifluorescence microscope.

### Ovule analysis of 35S:NTAP:AtCTF7∆B plants and wild type plants

Inflorescences from 35S:NTAP:AtCTF7∆B plants and wild type plants were collected and fixed in 4% glutaraldehyde under vacuum for 2 hrs, dehydrated in a graded ethanol series (40%, 60%, 80%, 100% steps for 1 h each), and cleared in a 2:1 mixture of benzyl benzoate:benzyl alcohol. Ovules were dissected under a stereo dissecting microscope, mounted and sealed with coverslips. Ovules were observed on a Zeiss Axioskop microscope under differential interference contrast microscopy optics using a 40 objective as described [32]. Images were collected and processed. Ovules were also observed by confocal laser scanning microscopy [34,35]. Images were collected and projected with Olympus Flouview 2.0 software (http://www.olympus-global.com/) and analyzed with Image Pro Plus (Media Cybernetics; http://www.mediacy.com). All pistils from individual inflorescences were dissected and the ovules stages were recorded [34,35].

### Availability of supporting data

The supporting data of this article are included with the article and its additional files.

## Additional file

Additional file 1: Figure S1. Schematic diagrams of wild type AtCTF7 and AtCTF7 constructs used in this study. **Table S1.** Transfer efficiency of 35S:NTAP:AtCTF7∆B mutants. **Figure S2.** SYN1 distribution pattern is not altered in 35S:NTAP:AtCTF7∆B male meiocytes. **Figure S3.** Female gametophyte development in wild type plants, revealed by differential interference contrast microscopy. **Figure S4.** Female gametophyte development in wild type plants, revealed by confocal laser scanning microscopy. **Table S2.** Female gametophyte development in wild type plants. **Table S3.** Female gametophyte development in 35S:NTAP:AtCTF7∆B plants. **Figure S5.** 35S:NTAP:AtCTF7∆B ovules exhibit various defects during meiosis and mitosis. **Figure S6.** Female gametophytes from Line 13 and Line 15 resemble those from Line 11. **Figure S7.** Transcript levels of select genes in female gametophyte development are increased in 35S:NTAP:AtCTF7∆B plants. **Figure S8.** Morphological alterations are associated with 35S:AtCTF7∆B and *atctf7-1* plants transformed with CTF7_pro_:AtCTF7∆B. **Table S4.** Primers used.

## Abbreviations

At: Arabidopsis thaliana
∆B: ∆299-345
SMC: Structural Maintenance of Chromosome
SCC: Sister Chromatid Cohesion
RBS: Roberts syndrome
qRT-PCR: Quantitative reverse transcription polymerase chain reaction
FG: Female Gametophyte
DIC: Differential interference contrast microscopy
MMC: Megaspore mother cell
FM: Functional megaspore
CLSM: Confocal laser scanning microscopy
EC: Egg cell
CC: Central cell
SN: Synergid nucleus
FML: Functional megaspore-like
DM: Degenerated megaspore
TEs: Transposable elements
AGO: ARGONAUTE
DAPI: 4,6-diamino-2-phenylindole dihydrochloride

## References

[1] Nasmyth K, Haering CH (2009). Cohesin: its roles and mechanisms. Ann Rev Genet.

[2] Brooker AS, Berkowitz KM (2014). The roles of cohesins in mitosis, meiosis, and human health and disease. Methods Mol Biol.

[3] Zamariola L, Tiang CL, De Storme N, Pawlowski W, Geelen D (2014). Chromosome segregation in plant meiosis. Front Plant Sci.

[4] Watanabe Y, Nurse P (1999). Cohesin Rec8 is required for reductional chromosome segregation at meiosis. Nature.

[5] Haering CH, Lowe J, Hochwagen A, Nasmyth K (2002). Molecular architecture of SMC proteins and the yeast cohesin complex. Mol Cell.

[6] Bernard P, Schmidt CK, Vaur S, Dheur S, Drogat J, Genier S (2008). Cell-cycle regulation of cohesin stability along fission yeast chromosomes. EMBO J.

[7] Díaz-Martínez LA, Giménez-Abián JF, Clarke DJ (2008). Chromosome cohesion-rings, knots, orcs and fellowship. J Cell Sci.

[8] Yuan L, Yang X, Makaroff CA (2011). Plant cohesins, common themes and unique roles. Cur Protein and Peptide Sci.

[9] Tomonaga T, Nagao K, Kawasaki Y, Furuya K, Murakami A, Morishita J (2000). Characterization of fission yeast cohesin: essential anaphase proteolysis of Rad21 phosphorylated in the S phase. Genes Dev.

[10] Ben-Shahar TR, Heeger S, Lehane C, East P, Flynn H, Skehel M (2008). Eco1-dependent cohesin acetylation during establishment of sister chromatid cohesion. Science.

[11] Huang X, Andreu-Vieyra CV, Wang M, Cooney AJ, Matzuk MM, Zhang P (2009). Preimplantation mouse embryos depend on inhibitory phosphorylation of separase to prevent chromosome missegregation. Mol Cell Biol.

[12] Almedawar S, Colomina N, Bermúdez-López M, Pociño-Merino I, Torres-Rosell J (2012). A SUMO-dependent step during establishment of sister chromatid cohesion. Curr Biol.

[13] Wu N, Kong X, Ji Z, Zeng W, Potts PR, Yokomori K (2012). Scc1 sumoylation by Mms21 promotes sister chromatid recombination through counteracting Wapl. Genes Dev.

[14] Skibbens RV, Corson LB, Koshland D, Hieter P (1999). Ctf7p is essential for sister chromatid cohesion and links mitotic chromosome structure to the DNA replication machinery. Genes Dev.

[15] Toth A, Ciosk R, Uhlmann F, Galova M, Schleifer A, Nasmyth K (1999). Yeast cohesin complex requires a conserved protein, Eco1p (Ctf7), to establish cohesion between sister chromatids during DNA replication. Genes Dev.

[16] Jiang L, Yuan L, Xia M, Makaroff CA (2010). Proper levels of the Arabidopsis cohesion establishment factor CTF7 are essential for embryo and megagametophyte, but not endosperm, development. Plant Physiol.

[17] Bolanos-Villegas P, Yang X, Wang H, Juan C, Chuang M, Makaroff CA (2013). Arabidopsis CHROMOSOME TRANSMISSION FIDELITY 7 (AtCTF7/ECO1) is required for DNA repair, mitosis and meiosis. Plant J.

[18] Singh DK, Andreuzza S, Panoli AP, Siddiqi I (2013). AtCTF7 is required for establishment of sister chromatid cohesion and association of cohesin with chromatin during meiosis in Arabidopsis. BMC Plant Biol.

[19] Heidinger-Pauli JM, Unal E, Guacci V, Koshland D (2008). The kleisin subunit of cohesin dictates damage-induced cohesion. Mol Cell.

[20] Unal E, Heidinger-Pauli JM, Kim W, Guacci V, Onn I, Gygi SP (2008). A molecular determinant for the establishment of sister chromatid cohesion. Science.

[21] Whelan G, Kreidl E, Wutz G, Egner A, Peters JM, Eichele G (2012). Cohesin acetyltransferase Esco2 is a cell viability factor and is required for cohesion in pericentric heterochromatin. EMBO J.

[22] Tanaka K, Yonekawa T, Kawasaki Y, Kai M, Furuya K, Iwasaki M (2000). Fission yeast eso1p is required for establishing sister chromatid cohesion during S phase. Mol Cell Biol.

[23] Williams BC, Garrett-Engele CM, Li Z, Williams EV, Rosenman ED, Goldberg ML (2003). Two putative acetyltransferases, San and Deco, are required for establishing sister chromatid cohesion in Drosophila. Curr Biol.

[24] Hou F, Zou H (2005). Two human orthologues of Eco1/Ctf7 acetyltransferases are both required for proper sister-chromatid cohesion. Mol Biol Cell.

[25] Gard S, Light W, Xiong B, Bose T, McNairn AJ, Harris B (2009). Cohesinopathy mutations disrupt the subnuclear organization of chromatin. J Cell Biol.

[26] Vega H, Waisfisz Q, Gordillo M, Sakai N, Yanagihara I, Yamada M (2005). Roberts syndrome is caused by mutations in Esco2, a human homolog of yeast Eco1 that is essential for the establishment of sister chromatid cohesion. Nat Genet.

[27] Vega H, Trainer AH, Gordillo M, Crosier M, Kayserili H, Skovby F (2010). Phenotypic variability in 49 cases of ESCO2 mutations, including novel missense and codon deletion in the acetyltransferase domain, correlates with ESCO2 expression and establishes the clinical criteria for Roberts syndrome. J Med Genet.

[28] Dyda F, Klein DC, Hickman AB (2000). GCN5-related N-acetyltransferases: a structural overview. Annu Rev Biophys Biomol Struct.

[29] Ivanov D, Schleiffer A, Eisenhaber F, Mechtler K, Christian H, Nasmyth K (2002). Eco1 is a novel acetyltransferase that can acetylate proteins involved in cohesion. Curr Biol.

[30] Rohila JS, Chen M, Cerny R, Fromm ME (2004). Improved tandem affinity purification tag and methods for isolation of protein heterocomplexes from plants. Plant J.

[31] Cai X, Dong FG, Edelmann RE, Makaroff CA (2003). The Arabidopsis SYN1 cohesin protein is required for sister chromatid arm cohesion and homologous chromosome pairing. J Cell Sci.

[32] Schneitz K, Hulskamp M, Pruitt RE (1995). Wild-type ovule development in *Arabidopsis thaliana*-a light microscope study of cleared whole-mount tissue. Plant J.

[33] Olmedo-Monfil V, Durán-Figueroa N, Arteaga-Vázquez M, Demesa-Arévalo E, Autran D, Grimanelli D (2010). Control of female gamete formation by a small RNA pathway in Arabidopsis. Nature.

[34] Christensen CA, King EJ, Jordan JR, Drews GN (1997). Megagametogenesis in Arabidopsis wild type and the Gf mutant. Sex Plant Reprod.

[35] Christensen CA, Subramanian S, Drews GN (1998). Identification of gametophytic mutations affecting female gametophyte development in Arabidopsis. Dev Biol.

[36] Siddiqi I, Ganesh G, Grossniklaus U, Subbiah V (2000). The dyad gene is required for progression through female meiosis in Arabidopsis. Development.

[37] Barrell PJ, Grossniklaus U (2005). Confocal microscopy of whole ovules for analysis of reproductive development: the elongate1 mutant affects meiosis II. Plant J.

[38] Gross-Hardt R, Lenhard M, Laux T (2002). WUSCHEL signaling functions in interregional communication during Arabidopsis ovule development. Genes Dev.

[39] Yang X, Makaroff CA, Ma H (2003). The Arabidopsis MALE MEIOCYTE DEATH1 gene encodes a PHD-finger protein that is required for male meiosis. Plant Cell.

[40] Grelon M, Vezon D, Gendrot G, Pelletier G (2001). AtSPO11-1 is necessary for efficient meiotic recombination in plants. EMBO J.

[41] Higgins JD, Sanchez-Moran E, Armstrong SJ, Jones GH, Franklin FCH (2005). The Arabidopsis synaptonemal complex protein ZYP1 is required for chromosome synapsis and normal fidelity of crossing over. Genes Dev.

[42] Couteau F, Belzile F, Horlow C, Grandjean O, Vezon D (1999). Random chromosome segregation without meiotic arrest in both male and female meiocytes of a dmc1 mutant of Arabidopsis. Plant Cell.

[43] Jiang L, Xia M, Strittmatter LI, Makaroff CA (2007). The Arabidopsis cohesin protein SYN3 localizes to the nucleolus and is essential for gametogenesis. Plant J.

[44] Yuan L, Yang X, Ellis JL, Fisher NM, Makaroff CA (2012). The Arabidopsis SYN3 cohesin protein is important for early meiotic events. Plant J.

[45] d’Erfurth I, Jolivet S, Froger N, Catrice O, Novatchkova M (2009). Turning meiosis into mitosis. PLoS Biol.

[46] Moissiard G, Cokus S, Cary J, Feng S, Billi AC, Stroud H (2012). MORC Family ATPases Required for Heterochromatin Condensation and Gene Silencing. Science.

[47] Tian L, Chen ZJ (2001). Blocking histone deacetylation in *Arabidopsis* induces pleiotropic effects on plant gene regulation and development. Proc Natl Acad Sci U S A.

[48] Chen X (2009). Small RNAs and their roles in plant development. Annu Rev Cell Dev Biol.

[49] Matzke M, Kanno T, Daxinger L, Huettel B, Matzke AJ (2009). RNA-mediated chromatin-based silencing in plants. Curr Opin Cell Biol.

[50] Law JA, Jacobsen SE (2010). Establishing, maintaining and modifying DNA methylation patterns in plants and animals. Nat Rev Genet.

[51] Shi CL, Stenvik GE, Vie AK, Bones AM, Pautot V, Proveniers M (2011). Arabidopsis class I KNOTTED-like homeobox proteins act downstream in the IDA-HAE/HSL2 floral abscission signaling pathway. Plant Cell.

[52] Brands A, Skibbens RV (2005). Ctf7p/Eco1p exhibits acetyltransferase activity–but does it matter?. Curr Biol.

[53] Van Den Berg DJ, Francke U (1993). Roberts syndrome, a review of 100 cases and a new rating system for severity. Am J Med Genet.

[54] Dorsett D, Merkenschlager M (2013). Cohesin at active genes: a unifying theme for cohesin and gene expression from model organisms to humans. Curr Opin Cell Bio.

[55] Cromer L, Jolivet S, Horlow C, Chelysheva L, Heyman J, De Jaeger G (2013). Centromeric cohesion is protected twice at meiosis, by SHUGOSHINs at anaphase I and by PATRONUS at interkinesis. Curr Biol.

[56] Lu S, Goering M, Gard S, Xiong B, McNairn AJ, Jaspersen SL (2010). Eco1 is important for DNA damage repair in *S. cerevisiae*. Cell Cycle.

[57] Wuest SE, Vijverberg K, Schmidt A, Weiss M, Gheyselinck J, Lohr M (2010). Arabidopsis female gametophyte gene expression map reveals similarities between plant and animal gametes. Curr Biol.

[58] Schmidt A, Wuest SE, Vijverberg K, Baroux C, Kleen D, Grossniklaus U (2011). Transcriptome analysis of the Arabidopsis megaspore mother cell uncovers the importance of RNA helicases for plant germline development. PLoS Biol.

[59] Shi DQ, Yang WC (2011). Ovule development in Arabidopsis: progress and challenge. Curr Opin Plant Biol.

[60] Nonomura K, Morohoshi A, Nakano M, Eiguchi M, Miyao A, Hirochik H (2007). A germ cell–specific gene of the ARGONAUTE family is essential for the progression of premeiotic mitosis and meiosis during sporogenesis in rice. Plant Cell.

[61] Bose T, Lee KK, Lu S, Xu B, Harris B (2012). Cohesin proteins promote ribosomal RNA production and protein translation in yeast and human cells. PLoS Genet.

[62] Shi DQ, Liu J, Xiang YH, Ye D, Sundaresan V, Yang WC (2005). *SLOW WALKER1*, essential for gametogenesis in Arabidopsis, encodes a WD40 protein involved in 18S ribosomal RNA biogenesis. Plant Cell.

[63] Coury D, Zhang C, Ko A, Skaggs M, Christensen C, Drews GN (2007). Segregation distortion in Arabidopsis gametophytic factor 1 (gfa1) mutants is caused by a deficiency of an essential RNA splicing factor. Sex Plant Reprod.

[64] Groß-Hardt R, Kagi C, Baumann N, Moore JM, Baskar R, Gagliano WB (2007). LACHESIS restricts gametic cell fate in the female gametophyte of Arabidopsis. PLoS Biol.

[65] Huang CK, Huang LF, Huang JJ, Wu SJ, Yeh CH, Lu CA (2010). A DEAD-Box protein, AtRH36, is essential for female gametophyte development and is involved in rRNA biogenesis in Arabidopsis. Plant Cell Physiol.

[66] Zsögön A, Szakonyi D, Shi X, Byrne ME (2014). Ribosomal protein RPL27a promotes female gametophyte development in a dose-dependent manner. Plant Physio.

[67] Van Lijsebettens M, Vanderhaeghen R, De Block M, Bauw G, Villarroel R, Van Montagu M (1994). An S18 ribosomal protein gene copy at the Arabidopsis PFL locus affects plant development by its specific expression in meristems. EMBO J.

[68] Byrne ME, Simorowski J, Martienssen RA (2002). ASYMMETRIC LEAVES1 reveals knox gene redundancy in Arabidopsis. Development.

[69] Stirnberg P, Liu JP, Ward S, Kendall SL, Leyser O (2012). Mutation of the cytosolic ribosomal protein-encoding RPS10B gene affects shoot meristematic function in Arabidopsis. BMC Plant Biol.

[70] Clough SJ, Bent AF (1998). Floral dip: a simplified method for Agrobacterium-mediated transformation of *Arabidopsis thaliana*. Plant J.

[71] Alexander P (1969). Differential staining of aborted and nonaborted pollen. Stain Technol.

[72] Ross KJ, Fransz P, Jones GH (1996). A light microscopic atlas of meiosis in *Arabidopsis thaliana*. Chromosome Res.

