# Supplementary material for: Overexpression of a truncated CTF7 construct leads to pleiotropic defects in reproduction and vegetative growth in Arabidopsis
Source: BMC Plant Biol. 2015 Mar 5;15:74. doi: 10.1186/s12870-015-0452-2 (PMC4359560; doi:10.1186/s12870-015-0452-2)
Supplement: Additional file 1: Figure S1. — Schematic diagrams of wild type AtCTF7 and AtCTF7 constructs used in this study. Table S1. Transfer efficiency of 35S:NTAP:AtCTF7∆B mutants. Figure S2. SYN1 distribution pattern is not altered in 35S:NTAP:AtCTF7∆B male meiocytes. Figure S3. Female gametophyte development in wild type plants, revealed by differential interference contrast microscopy. Figure S4. Female gametophyte development in wild type plants, revealed by confocal laser scanning microscopy. Table S2. Female gametophyte development in wild type plants. Table S3. Female gametophyte development in 35S:NTAP:AtCTF7∆B plants. Figure S5. 35S:NTAP:AtCTF7∆B ovules exhibit various defects during meiosis and mitosis. Figure S6. Female gametophytes from Line 13 and Line 15 resemble those from Line 11. Figure S7. Transcript levels of select genes in female gametophyte development are increased in 35S:NTAP:AtCTF7∆B plants. Figure S8. Morphological alterations are associated with 35S:AtCTF7∆B and atctf7-1 plants transformed with CTF7pro:AtCTF7∆B. Table S4. Primers used. [file 12870_2015_452_MOESM1_ESM.pdf]

Additional file

Overexpression of a truncated CTF7 construct leads to pleiotropic defects in reproduction and vegetative growth in Arabidopsis

Desheng Liu and **Christopher A. Makaroff***

Department of Chemistry and Biochemistry, Miami University, Oxford, OH 45056, United States of America

*Author for correspondence

Christopher A. Makaroff,

Tel: phone: +513 529 2813,

Fax :+513 529 5715,

e-mail: makaroca@miamioh.edu

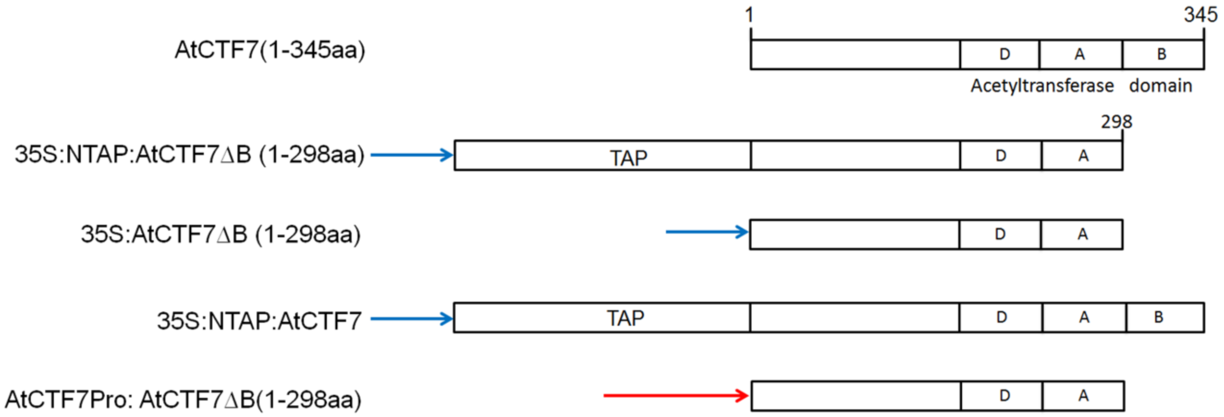

Figure S1. Schematic diagrams of wild type AtCTF7 and AtCTF7 constructs used in this study. Blue arrow represents 35 promoter and red arrow represents AtCTF7 promoter.

Table S1. Transfer efficiency of 35S:NTAP:AtCTF7ΔB mutants

Female (♀) X male(♂)	Basta Resistant	Basta Sensitive	Seeds not germinate
WT X Line 11	137 (38.3%)	188 (52.5%)	33 (9.3%)
Line 11 X WT	71 (25%)	124 (43.7%)	89 (31.3%)
Line 11 self-pollinated	354 (30.4%)	49 (4.2%)	763 (65.4%)

Line 11 plants were used in the crosses.

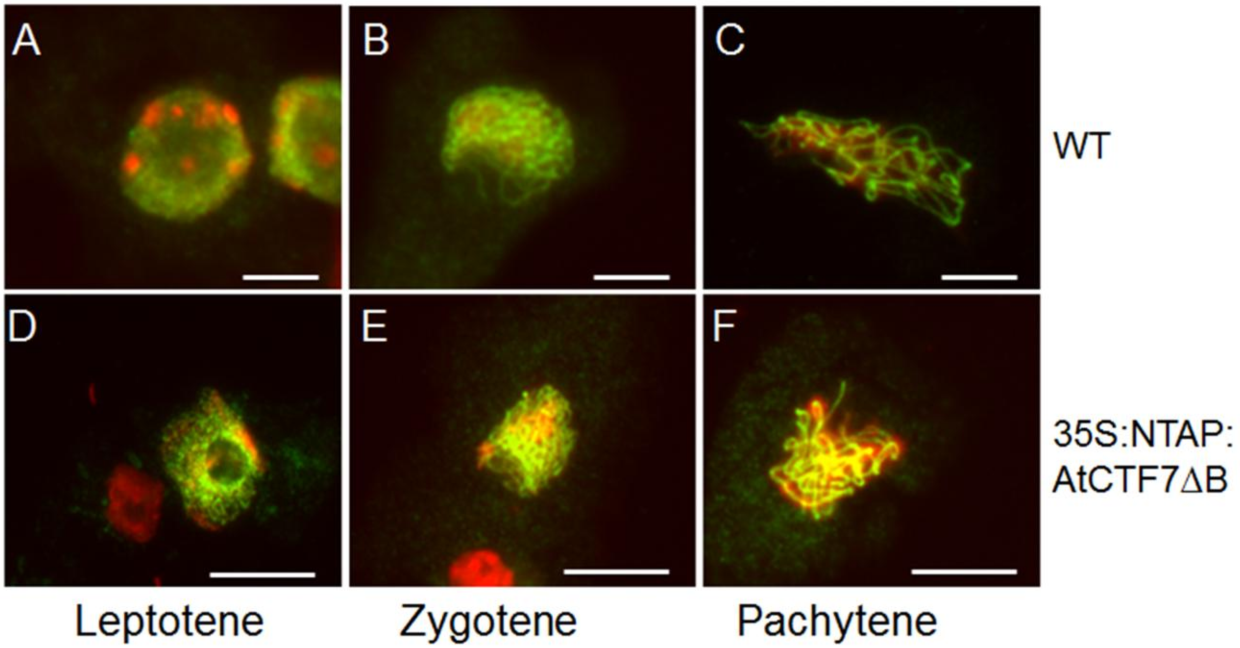

Figure S2. SYN1 distribution pattern is not altered in 35S:NTAP:AtCTF7ΔB male meiocytes. A to C, Meiocytes from wild type plants. D to F, Meiocytes from 35S:NTAP:AtCTF7ΔB plants. Interphase/early leptotene (A, D); zygotene (B, E), pachytene (C, F). Merged images of 4′,6-diamidino-2-phenylindole (DAPI) stained chromosomes (red) and SYN1 (green) are shown. Scale bar =10 μm.

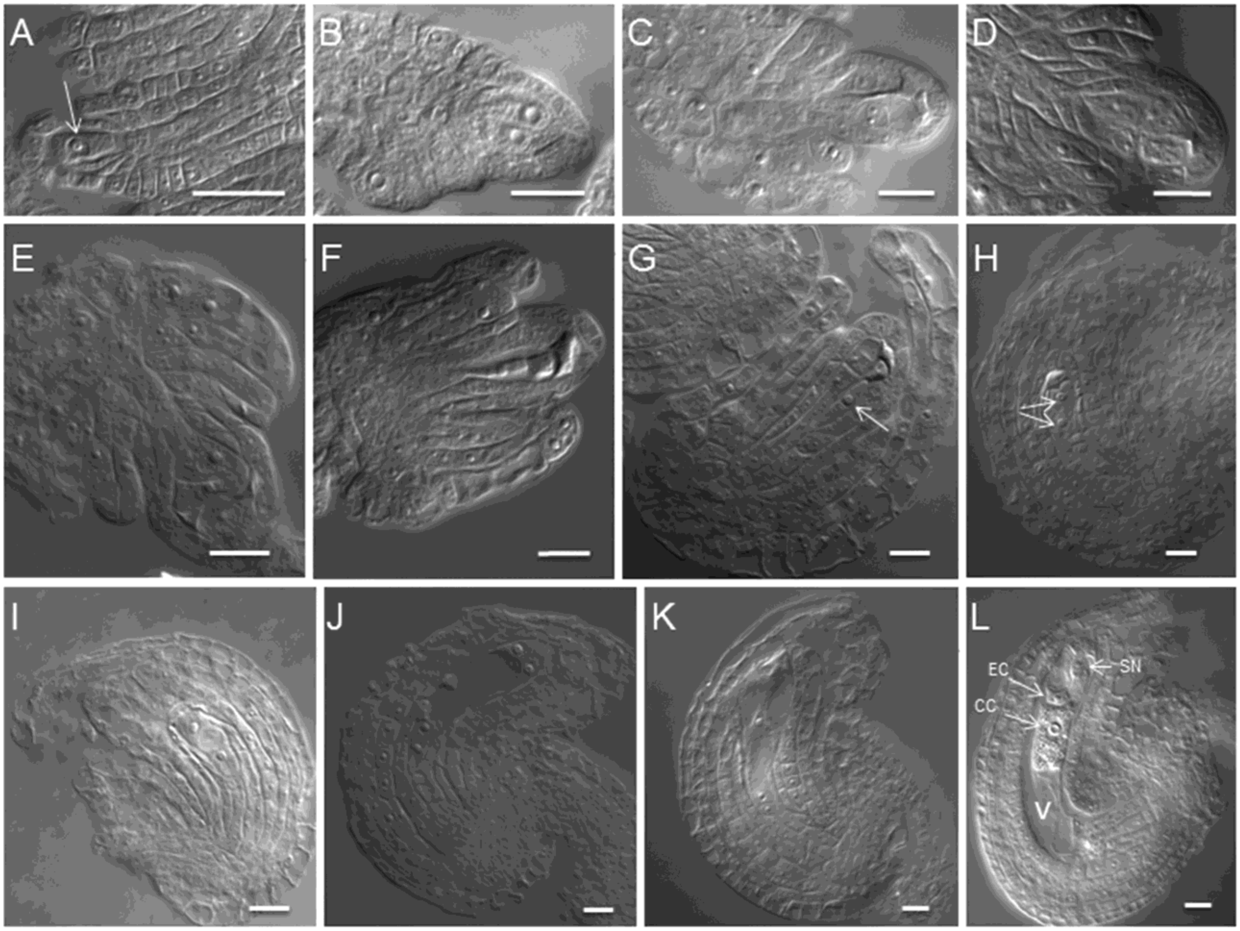

Figure S3. Female gametophyte development in wild type plants, revealed by differential interference contrast (DIC) microscopy. A, Pre-meiotic ovule at stage 1-II. Arrow denotes the megaspore mother cell (MMC). B, Meiotic ovule at stage 2-IV containing two longitudinally megaspore cells (dyad) after meiosis I. C, Meiotic ovule at stage 2-IV containing a dyad and the bottom megaspore containing two nuclei. D, Meiotic ovule at stage 2-V containing a tetrad after meiosis II. E, Meiotic ovule at stage 2-V containing a linear tetrad. F, Early Female Gametophyte stage 1 (FG1) ovule (stage 3-I). G, FG1 ovule (stage 3-I). FM (arrow) is uni-nucleate and there is still space at the distal end of the ovule. H, FG2 ovule containing two nuclei (arrows) (stage 3-II). I, FG3 ovule containing a two-nucleate embryo sac with a central vacuole (stage 3-III). J, FG4 ovule containing a four-nucleate embryo sac (stage 3-IV). K, FG5 ovule after cell

differentiation and cellularization (stage 3-V). L, FG7 ovule (stage 3-VI). A diploid central cell (CC) is formed and the antipodal cells (ANs) are degenerated. CC, central cell; EC, egg cell; MMC, megaspore mother cell; SN, synergid nucleus; V, vacuole. Scale bar=10 μm. Ovule stages were determined according to Schneitz et al. [32].

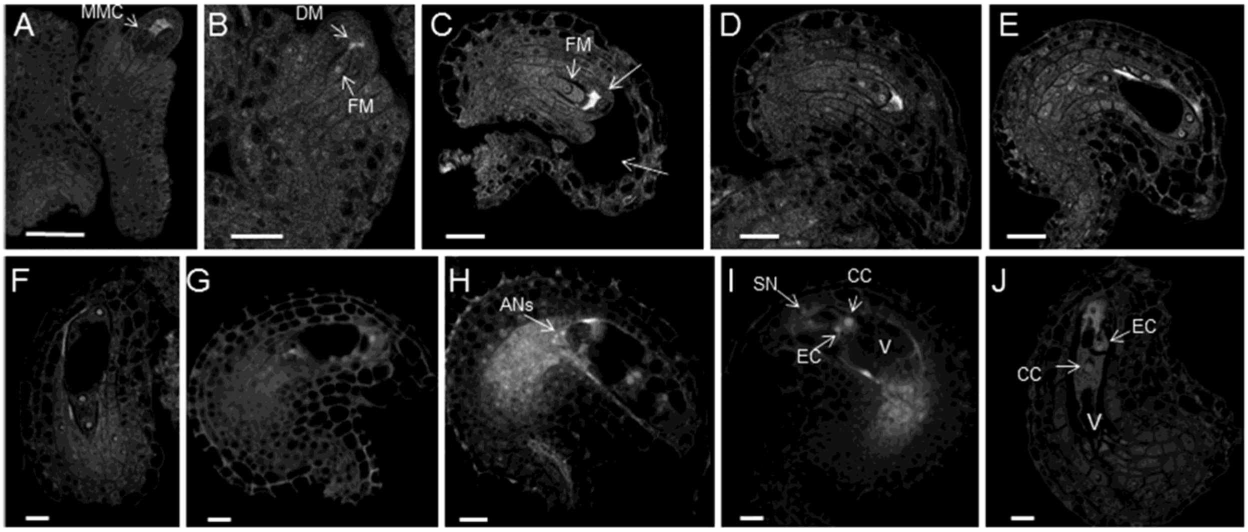

Figure S4. Female gametophyte development in wild type plants, revealed by confocal laser scanning microscopy (CLSM). A, FG0 ovule. The MMC is surrounded by the nucellar epidermis. The outer and inner integuments just start to initiate. B, Early FG1 ovule containing FM and DM. The nucellus is not surrounded by the integuments. C, FG1 ovule. The nucellus is surrounded by the outer integuments but not the inner integuments. The space is not filled by the inner integuments (arrow). D, FG3 ovule containing a two-nucleate embryo sac. The two nuclei are separated by a central vacuole and the nucellus is enclosed by the inner integument. E, Early FG4 ovule containing a four-nucleate embryo sac. The division planes of the two pair nuclei are orthogonal to each other. F, Late FG4 ovule. A vacuole appears at the chalazal end of the embryo sac. G, FG5 ovule. The embryo sac contains eight nuclei in a 4n+4n configuration. Not all the nuclei are visible on this plane. H, FG6 ovule after the cellularization. Three ANs are degenerating (arrow) and CC does not form. I, FG7 ovule containing a four-nuclei embryo sac. CC forms and ANs have degenerated. J, FG8 ovule prior to fertilization. The embryo sac consists of EC and CC. AN, antipodal nucleus; CC, central cell; DM, degenerated megaspore; EC, egg cell; FM, functional megaspore; MMC, megaspore mother cell; SN, synergid nucleus; V,

vacuole. Bar scale=10 μm. Ovule developmental stages were defined according to Christensen et al. [34] [35].

Table S2. Female gametophyte development in wild type (Col) plants

Pistil #	FG1	FG2	FG3	FG4	FG5	FG6	FG7	FG8
1	30							
2	1	2	26					
3			4	23				
4				18	13			
5				8	26	1		
6				1	7	38		
7					1	7	16	15
8							1	42

Developmental stages are defined according to Christensen et al. (1997, 1998)[34][35].

Table S3. Female gametophyte development in 35S:NTAP:AtCTF7ΔB plants

Pistil #	FG0	FG1	FG2	FG-A	FG3	FG4	FG5	FG6	FG7	FG-B	FG-C
1	25	1									
2	22	6	0	1							
3	15	10	0	2							
4	8	18	0	3							
5	1	25	0	1							
6		20	2	6							
7		4	15	2	6						
8			2	4	20						
9			1	0	16	1	2	0	0	0	3
10			1	0	17	2	0	0	0	0	8
11			3	0	10	0	0	3	0	0	13
12			12	3	0	0	0	0	1	0	18
13			8	0	1	0	0	0	0	2	20
14			4	0	0	0	0	0	0	12	15

A 4th generation Line 11 plant was used.

FG0, Ovules before FG1 (Fig. 4F; Supplemental Fig. S5, A, B, E and F); FG-A, Small ovules with no observable nuclei (Supplemental Fig. S5D); FG-B, Terminal ovules without embryo sac (Supplemental Fig. S5M); FG-C, Terminal ovules with nuclei (Supplemental Fig. S5, N-P). Female gametophyte stages are defined according to Christensen et al. (1997, 1998) [34],[35.]

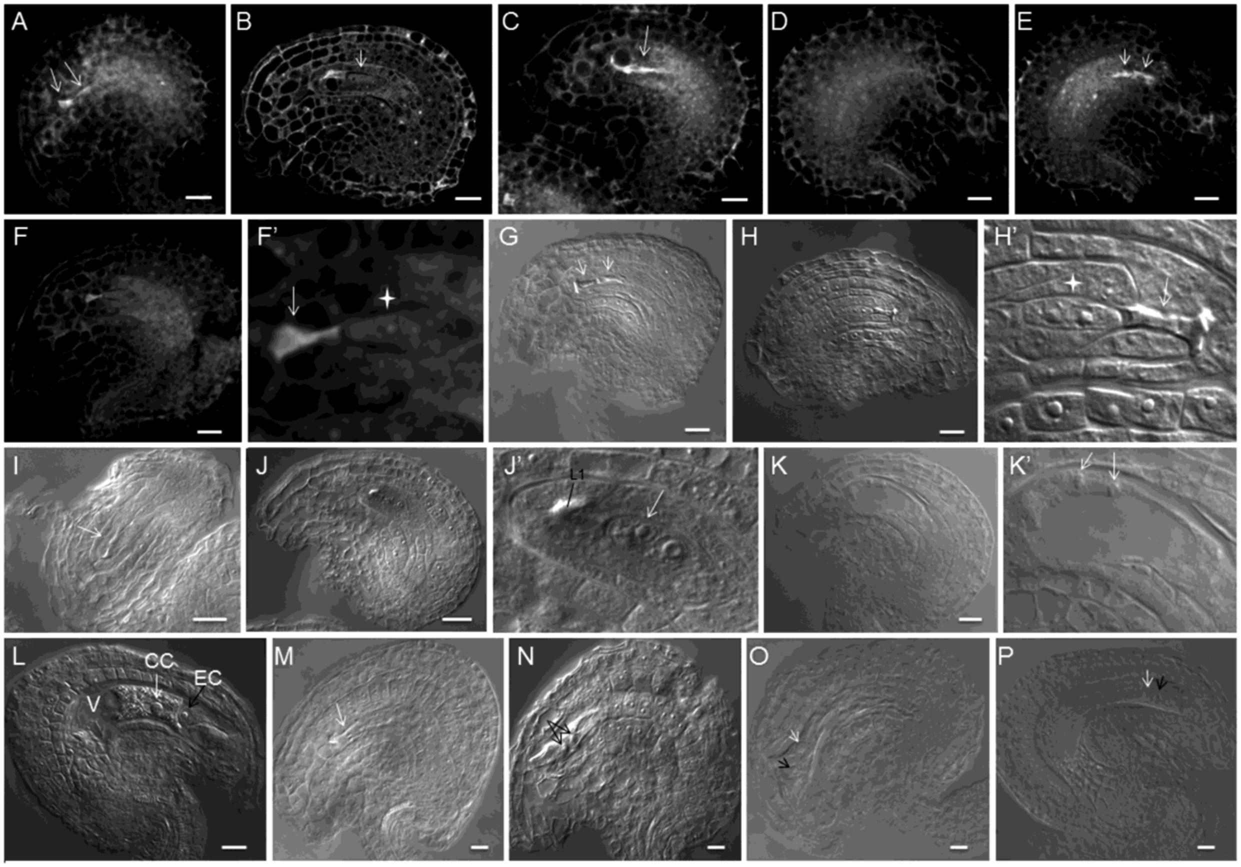

Figure S5. 35S:NTAP:AtCTF7ΔB ovules exhibit various defects during meiosis and mitosis. A, Ovule containing two degrading megaspores. B, Ovule containing a degenerated megaspore (DM) and anucleate megaspore (arrow), indicating the nucleus is completely degraded. C, Ovule containing a mis-shaped functional megaspore-like (FML) cell with a degrading nucleus (arrow). D, Ovule containing no visible megaspore(s), indicating the megaspore(s) are completely degraded. E, Two elongated cells with no defined nuclei but diffuse signal throughout the cells (arrows). F&F', DM at the distal position (arrow) and a chalazal end cell (star) containing multiple nuclei. Nuclei are not separated by a cell wall. G, Two elongated cells (arrows). H&H',, Cell at the chalazal end contains two nuclei (star). I to K, DIC showing degenerating megaspores/embryo sacs during mitosis. I, FG1 ovule containing a FML-cell (arrow). J&J', Embryo sac containing one degenerating nucleus (arrow) and one surviving nucleus. Non-

degenerated L1 layer is marked with a star. K&K’, Ovule containing two degrading nuclei (arrows) on the side of the vacuole. Other nuclei are not visible. Ovule resembles FG5 stage. L, Wild type ovule at FG7. CC and EC are marked. M to P, Terminal 35S:NTAP:AtCTF7ΔB ovules containing various defective embryo sacs by DIC. M, Ovule containing the debris of an embryo sac (arrow). N, Embryo sac containing two nuclei (arrows), morphologically similar to EC. No vacuole is present. O, Embryo sac containing EC (black arrow) and CC (white arrow). EC and CC are not separated. No vacuole is present. P, Embryo sac containing EC (black arrow) and CC (white arrow). EC and CC are not separated. A large vacuole is present at the basal end. FMLs and DMs are identified according to Barrell and Grossniklaus [37]. Bar scale=10 μm. Ovule stages are determined according to Schneitz et al in DIC [32] and to Christensen in CLSM [34],[35].

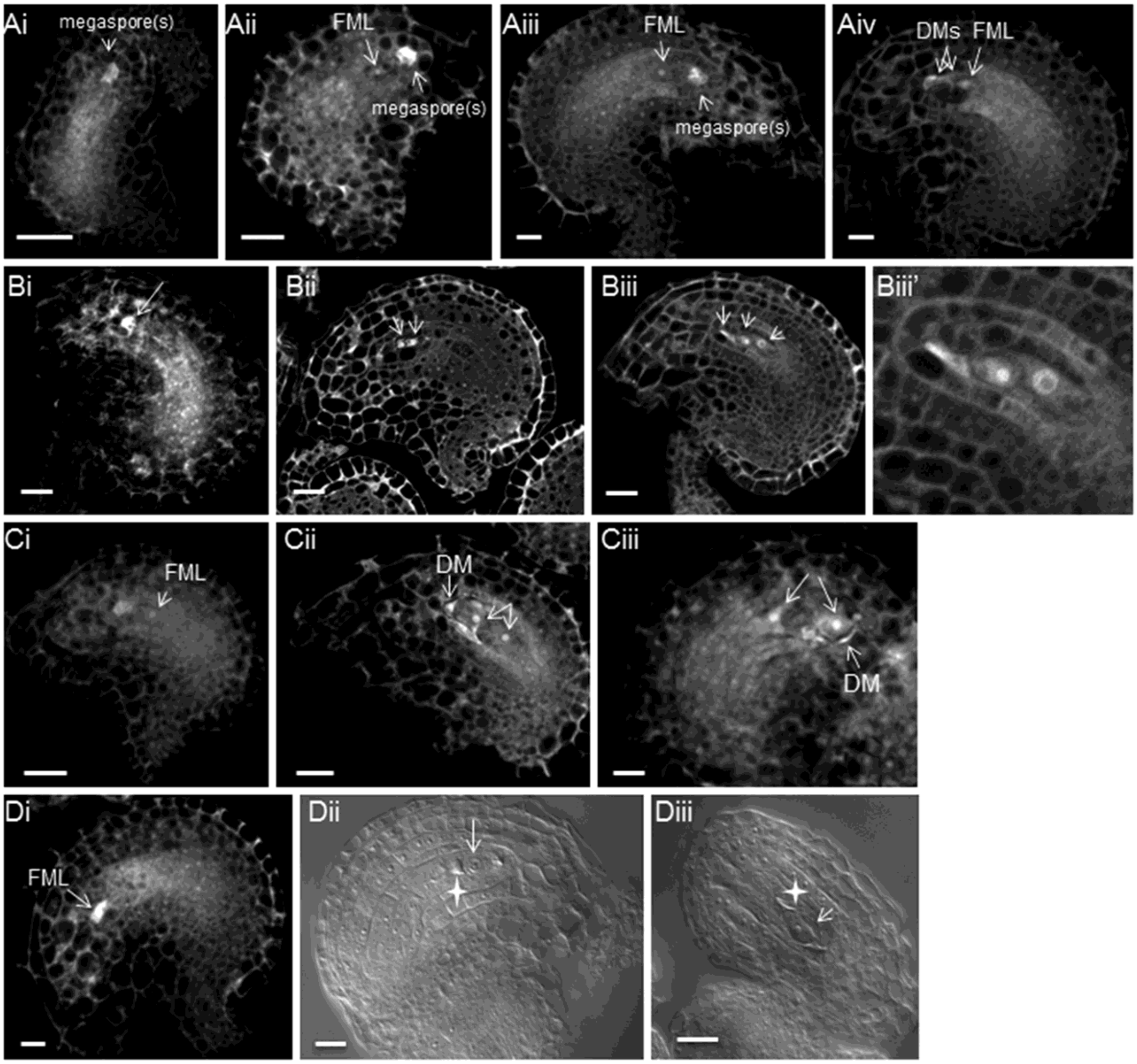

Figure S6. Female gametophytes from Lines 13 and Line 15 resemble those from Line 11. A, Female gametophytes from Line 13 develop slowly. Ai, Ovule containing clustered megaspores. Aii to Aiii, Ovules containing FMLs (arrows) and clustered megaspores (arrows). A megaspore separates from the clustered megaspores and becomes a FML at chalazal end. Aiv, Ovule containing a FML and two DMs. B, Two FMLs co-exist in ovules of Line 13. Bi, Ovule containing megaspore(s) with bright fluorescence signal (arrow). Bii, Two cells with strong fluorescence. Biii,iii', Two cells with highly stained nuclei at the chalazal end, resembling FMLs.

They are clearly separated and the right has enlarged nucleus. iii’, Magnified view of iii. C, Female gametophytes from Line 15 develop slowly. Ci, Ovule containing a FML (arrow) and clustered megaspores. Cii to Ciii, FG3 ovules. The separated megaspore at chalazal-end becomes a FML. FML completes one round of mitosis to produce two nuclei and a vacuole forms between them. D, Middle megaspores become FMLs in ovules of Line 15. Di, Ovule containing only a FML (arrow). Its nucleus is brightly stained but no associated DM(s) is identified. Dii to Diii, Middle megaspores (arrows) become FMLs while the associated megaspores (stars) degrade. DM, degenerated megaspore; FML, functional megaspore like. Bar scale=10 μm. FMLs are identified as distinctly bright autofluorescence in the nuclei and DMs containing a diffuse signal throughout the cells but no clearly defined nucleus, as defined according to Barrell and Grossniklaus [37]. Ovule stages are determined according to Schneitz et al. in DIC [32] and Christensen et al. in CLSM [34],[35].

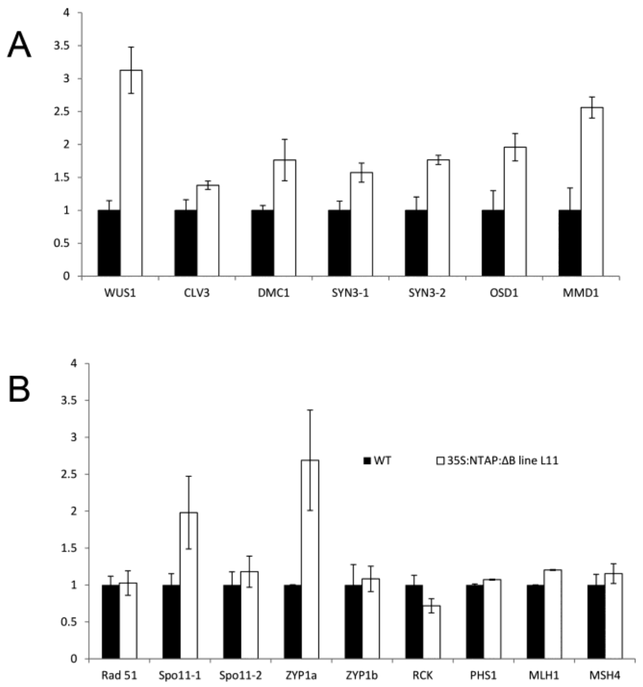

Figure S7. Transcript levels of genes in female gametophyte development and recombination are increased in 35S:NTAP:AtCTF7ΔB plants. Buds of wild type, and 4th generation 35S:NTAP:AtCTF7ΔB Line 11 plants were the source of material. Data are shown as means ± SD (*n* = 3).

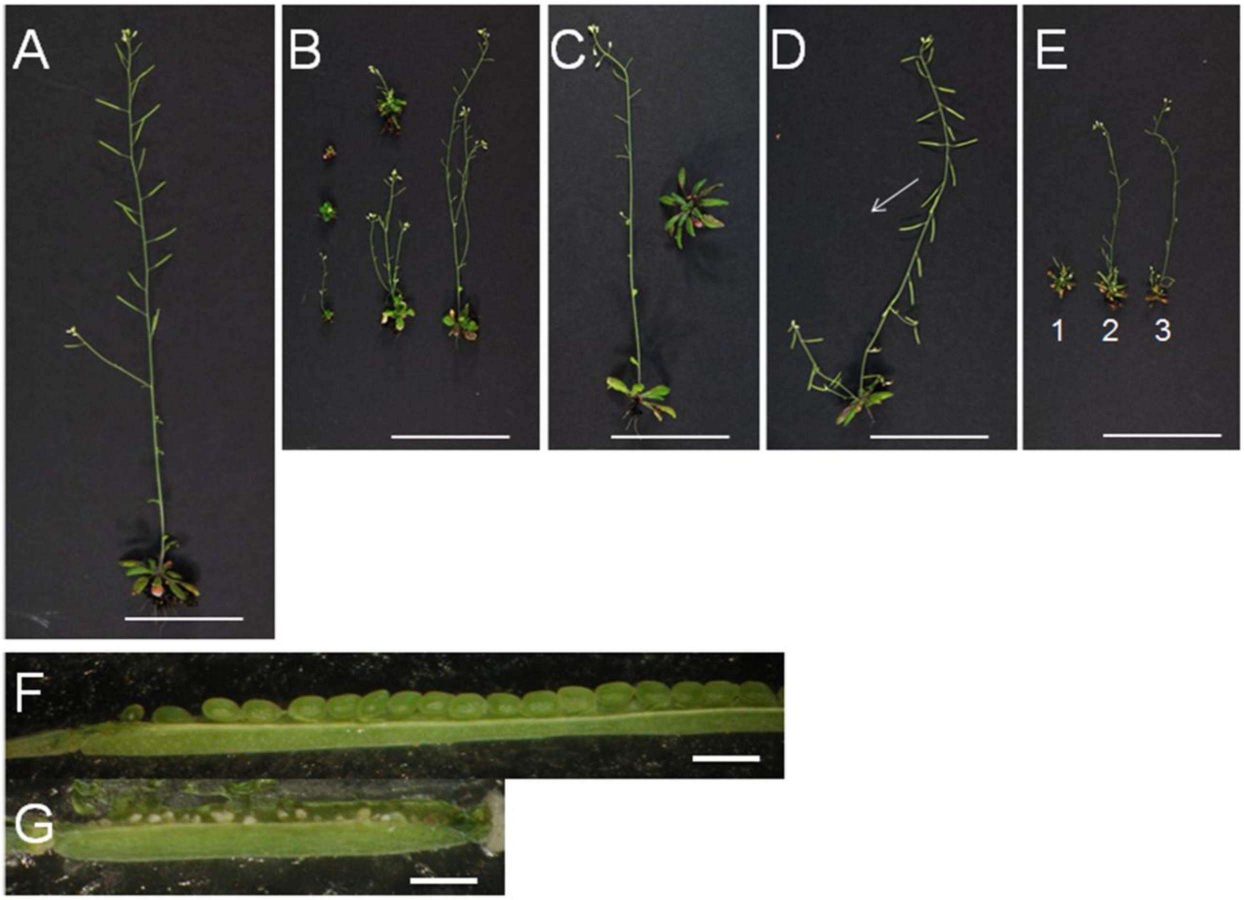

Figure S8. Morphological alterations are associated with 35S:AtCTF7ΔB and *atctf7-1* plants transformed with CTF7$_{pro}$:AtCTF7ΔB. A, Wild type plant. B, 35S:AtCTF7ΔB plants from Line 21. Dwarf plants appear at a high frequency with varying severity. C, 35S:AtCTF7ΔB plants from Line 24. "Normal" reduced fertile plants and plants without an inflorescence are present in the same progeny. D, 35S:AtCTF7ΔB plant from Line 29 containing multiple inflorescences with downward-pointing siliques. E, Homozygous *atctf7-1* plants containing CTF7$_{pro}$:AtCTF7ΔB construct. All plants are dwarf. Some plants (E1) lack stems and some plants (E3) produce reduced numbers of rosette leaves. F, Wild type silique with full seed set. G, Silique from *atctf7-1*:CTF7$_{pro}$:AtCTF7ΔB plant containing about 40 aborted ovules. All plants

are 30-days old and grown under the same environmental conditions. All 35S:AtCTF7ΔB plants are 3rd generation. Scale bar= 5 cm in A-E. Scale bar=0.5 cm in F/G.

Table S4. Primers used

Primers	Sequence 5'-3'	Purpose
**Genotyping**		
SALK059500LP	ATGAATAGAAATGAGCGATAGGC	Genomic forward
SALK059500RP	GAAGCTATGAGCTTAAATGCTTCC	Genomic reverse
SALK LBb1.3	ATTTTGCCGATTTCGGAAC	Left border forward for SALK lines
**Cloning**		
AtCTF7ΔB gateway F	CACCATGCAAGCCAAAATCAATTC	Forward primer for entry clone of 35S:NTAP:CTF7ΔB
AtCTF7ΔB gateway R	GCTAGTTATTGCTCAGCGG	Reverse primer for entry clone of 35S:NTAP:CTF7ΔB
AtCTF7 ΔB F	GGAGCCATGGATATGCAAGCCAAAATC	Forward primer for 35S CTF7ΔB
AtCTF7 ΔB R	GAGACTAGTTTAATTCTAGTTATTGC	Reverse primer for 35S

	TCAGCGG	
AtCTF7 gateway F	CACCATGCAAGCCAAAATCAATTC	Forward primer for entry clone of 35S:NTAP:CTF7
AtCTF7 gateway R	TTAAGAAAAGTGAGTATCAATT	Reverse primer for entry clone of 35S:NTAP:CTF7
CTF7pro F	CCGGAATTCCACATCCT GGAAATATCTTTGCAA	Forward primer for CTF7promoter
CTF7pro R	GGAGCCATGGCGTCTAGAG AGAGCTCGAATCCTTGTT	Reverse primer for CTF7promoter
**qRT-PCR**		To quantify expression
Tubulin F	TGGATCATGAGTGAGTGAAAAGA	Expression control
Tubulin R	AAAACCACAATGGACAATTTC	
CTF7 native+O/E F	GTTGGGTGAGGATTGGAT TC	
CTF7 native+O/E R	GCGAGGATGAGCTCTCTTTT	
CTF7 native F	GTGGGATTAGAGCGATTTGG	
CTF7 native R	TTCCTATGGAGCTTGGTTGTG	
WUS F	TGGATCTATGGAACAAGACTGTT	
WUS R	GGCTTTGCTCTATCGAAGAAGT	
CLV3 F	CGAAGGGTTTAGGACTACATGAAG	
CLV3 R	GTGGGTTCACATGATGGTGCAA	
SYN3-1 F	AAAGAAATTTGGGGCTTTCA	
SYN3-1 R	TGGTGTTCCTACTGGGGAAT	
SYN3-2 F	CGAGTTCGACTTGGAAGATG	

SYN3-2 R	AAGGATCAATGCCAGTAGGAA	
DMC1 F	GTTCATATCAGACCCAAAAAAGCC	
DMC1 R	AGATTCGGAGCATCGTAGACTTTG	
OSD1 F	GAATCTCCGGTGAATCCAGA	
OSD1R	AGAAGGCAACAAACCACCAC	
MMD1 F	TATCCGCGGTATGACTGTGT	
MMD1 R	GCAATAGGGTTCCGATGAAT	
RBR F	GGTGGAGGAGAAACTTGTGC	
RBR R	GTGGTTGCTTCCGGTAGTTG	
MU F	TAATTTGGCTGACGGAATCAC	
MU R	ATTTGGGGGAAAACAAATGAG	
COPIA28 F	AGTCCTTTTGGTTGCTGAACA	
COPIA28 R	CCGGATGTAGCAACATTCACT	
SoloLTR F	AACTAACGTCATTACATACACATCTTG	
SoloLTR R	AATTAGGATCTTGTTTGCCAGCTA	
HDA19 F	GACTGTGATTACAACACACCGT	
HDA19 R	AATTGCCGCCAGTATCCAT	
AGO1 F	TGGACCACCGCAGAGACAAT	
AGO1 R	CATCATACGCTGGAAGACGACT	
AGO4 F	CACTCGCTCTCCTATGTGTACCAAAG	
AGO4 R	CATGGCTTGATGATGTCTCAGACTGATC	
RDR2 F	TGGCGAGAGATAACCGGAGGTATG	
RDR2 R	CTTCTCATCGCGATGGTTTGGATTG	

DCL3 F	GCCTACTTTCGATACCTCGGAAGA	
DCL3 R	GCATACATCACAGCCTCACGATTG	
NRPD1A F	GACTTGTGAAGATGGTTCTGCAGTTG	
NRPD1A R	GTCTTCGAATGTCCCGTCTATTCTTAC	
MIR 156 F	CTCTCCCTCCCTCTCTTTGATTC	
MIR 156 R	AGGCCAAAGAGATCAGCACCGG	
MIR 172 F	TTTCTCAAGCTTTAGGTATTTGTAG	
MIR 172R	TCGGCGGATCCATGGAAGAAAGCTC	
MET1 F	GTGATTCTTAGGGCTATAATGG	
MET1 R	CATTGATGAAGTCCACTTGAC	
DMT7 F	CCCACCTGAGTTTGTGGACT	
DMT7 R	CATTCTGGCCACCATCTCTT	
RDM4 F	ATGGATGGGGTGGGTGAAAG	
RDM4 R	TAGCACCTTCTTCGGTTTCAC	
